# HoloVir: A Workflow for Investigating the Diversity and Function of Viruses in Invertebrate Holobionts

**DOI:** 10.3389/fmicb.2016.00822

**Published:** 2016-06-09

**Authors:** Patrick W. Laffy, Elisha M. Wood-Charlson, Dmitrij Turaev, Karen D. Weynberg, Emmanuelle S. Botté, Madeleine J. H. van Oppen, Nicole S. Webster, Thomas Rattei

**Affiliations:** ^1^Australian Institute of Marine ScienceTownsville, QLD, Australia; ^2^Center for Microbial Oceanography: Research and Education, University of Hawai‘i at MānoaHonolulu, HI, USA; ^3^Division of Computational Systems Biology, Department of Microbiology and Ecosystem Science, University of ViennaVienna, Austria; ^4^School of Biosciences, University of MelbourneMelbourne, VIC, Australia

**Keywords:** viral metagenomics, marine invertebrates, symbiosis, host-associated communities, Bioinformatics tools, marine ecology

## Abstract

Abundant bioinformatics resources are available for the study of complex microbial metagenomes, however their utility in viral metagenomics is limited. HoloVir is a robust and flexible data analysis pipeline that provides an optimized and validated workflow for taxonomic and functional characterization of viral metagenomes derived from invertebrate holobionts. Simulated viral metagenomes comprising varying levels of viral diversity and abundance were used to determine the optimal assembly and gene prediction strategy, and multiple sequence assembly methods and gene prediction tools were tested in order to optimize our analysis workflow. HoloVir performs pairwise comparisons of single read and predicted gene datasets against the viral RefSeq database to assign taxonomy and additional comparison to phage-specific and cellular markers is undertaken to support the taxonomic assignments and identify potential cellular contamination. Broad functional classification of the predicted genes is provided by assignment of COG microbial functional category classifications using EggNOG and higher resolution functional analysis is achieved by searching for enrichment of specific Swiss-Prot keywords within the viral metagenome. Application of HoloVir to viral metagenomes from the coral *Pocillopora damicornis* and the sponge *Rhopaloeides odorabile* demonstrated that HoloVir provides a valuable tool to characterize holobiont viral communities across species, environments, or experiments.

## Introduction

Marine viruses are the most abundant biological entities in the oceans, often exceeding the number of bacteria 10-fold (Wommack and Colwell, 2000; Suttle, 2005, 2007) and having high local and global diversity (Brum et al., 2015). Viruses infect all living bacterial, archaeal and eukaryotic cells (Fuhrman, 1999; Wommack and Colwell, 2000; Danovaro et al., 2008; Rohwer and Thurber, 2009) and are responsible for high turnover rates of their microbial hosts and subsequent nutrient cycling in the world's oceans (Weitz and Wilhelm, 2012). However, viruses are not exclusively agents of mortality, and in some cases, they can form mutually beneficial partnerships with their hosts (Weinbauer and Rassoulzadegan, 2004). For instance, viruses can contribute to host survival by suspending unnecessary metabolic activities during unfavorable environmental conditions, while they modulate host metabolic gene expression (Paul, 2008) and confer host fitness (Roossinck, 2011).

Due to limitations in traditional methodologies used for studying complex viral populations, including a lack of suitable marker genes, and limited methods designed specifically for viruses, our understanding about the specific roles viruses play in marine ecosystems has lagged behind our knowledge of the functional roles of cellular microorganisms. However, despite these limitations, research over the past decade has shown that viruses play a vital role in biogeochemical cycles as they modulate microbially-driven processes through mortality and subsequent release of organic matter and inorganic nutrients that become available for other microorganisms to consume (Suttle, 2005; Weitz and Wilhelm, 2012). This viral-induced mortality can be selective, thereby determining host community composition and acting as an important bottom-up ecological driver in marine ecosystems (Bouvier and del Giorgio, 2007; Hewson and Fuhrman, 2007). Horizontal gene transfer and metabolic reprogramming by viral-encoded auxiliary metabolic genes is another important ecosystem role (Jiang and Paul, 1998; Hurwitz et al., 2015) with the recombination of viral and host genes during infection often triggering changes in host metabolism, immunity, distribution and evolution (Rohwer and Thurber, 2009) as well as shaping viral genomes (Lindell et al., 2007).

To date, most of the research exploring interactions between viruses and eukaryotes within the marine environment has focused on causative agents of disease in commercially important taxa (reviewed in Suttle, 2007). However, as our understanding of the critical importance of the holobiont (host and the associated microbiome; Rohwer et al., 2002) has matured, research has begun to concentrate on viral associations in other marine species including reef invertebrates such as anemones, scleractinian corals and their algal endosymbionts (Wilson and Chapman, 2001; Wilson et al., 2001, 2005; Marhaver et al., 2008; Vega Thurber et al., 2008; Correa et al., 2013; Hewson et al., 2014; Pollock et al., 2014; Soffer et al., 2014). Advances in metagenomic sequencing have greatly improved our ability to explore viral communities associated with marine invertebrates (Marhaver et al., 2008; Wilson, 2012; Soffer et al., 2014; Weynberg et al., 2014); however, understanding the biodiversity and functional roles of viruses in a holobiont ecosystem context is still extremely challenging.

Tools to analyse complex metagenomes have primarily been developed for microbial (cellular) data sets, as these have well established and curated databases and are not affected by some of the methodological limitations that pertain to viruses. For example, the preparation of viral nucleic acid for whole genome sequencing requires an amplification step prior to sequencing, which can limit our ability to quantify viral biodiversity, and potentially limits our capacity to use coverage information in metagenome binning strategies (Albertsen et al., 2013; Smits et al., 2015). Although there is some evidence for quantifiable amplification of viral metagenomes, the focus has been solely on double stranded DNA (dsDNA) viruses, particularly the bacteriophage (viruses that infect bacteria) which are known to dominate pelagic marine ecosystems (Duhaime and Sullivan, 2012; Solonenko and Sullivan, 2013; Brum and Sullivan, 2015). Holobiont viral communities however, are much more complex and contain a diverse array of dsDNA/single stranded DNA (ssDNA) bacteriophage as well as a range of viruses that infect eukaryotes (Marhaver et al., 2008; Vega Thurber et al., 2008; Hewson et al., 2012; Correa et al., 2013; Weynberg et al., 2014; Wood-Charlson et al., 2015). Many of the pioneering marine invertebrate metavirome studies suffered from limitations in sample preparation and virome bioinformatics that restricted the biological interpretation of the sequence datasets (reviewed in Wood-Charlson et al., 2015). Whilst recent methodological improvements for purifying, extracting, and sequencing DNA and RNA viromes have enhanced our ability to capture greater viral diversity from marine samples (Weynberg et al., 2014), the metagenomic exploration of invertebrate-associated viral assemblages is a relatively new field and the majority of viral sequences still have no identifiable homologs in sequence databases.

To accelerate progress in the field of holobiont viromics, we require a customized bioinformatic analysis workflow that determines both the composition and putative function of viruses associated with ecologically important marine species. Importantly, analyses should be performed at both read and contig levels, as previous simulation studies have indicated that sequence assembly of viromes can be incomplete and is limited by chimeric contigs that can occur at all taxonomic levels (Vázquez-Castellanos et al., 2014; Smits et al., 2015).

Several existing bioinformatic platforms, such as Integrated Microbial Genomes (IMG) (Markowitz et al., 2014), Metagenomics-Rapid Annotation using Subsystem Technology (MG-RAST) (Meyer et al., 2008), Cyberinfrastructure for Advanced Microbial Ecology Research and Analysis (CAMERA) (Sun et al., 2011), and iPlant (Goff et al., 2011), provide metagenomic sequence analysis tools; however, each has limitations that restrict their applicability for invertebrate-associated viral metagenomes. For example, IMG/M, the analysis portal for the Joint Genome Institute, Department of Energy, USA, does not currently accept external sequencing projects; MG-RAST relies on curated bacterial-focused databases that are inappropriate for viruses and CAMERA was discontinued in 2014. Since then, CAMERA's sequence data has been transferred to the iMicrobe portal (http://imicrobe.us/, supported by iPlant) and although iMicrobe, and its cousin iVirus (still under development), are likely to be valuable resources, they are not currently funded to accommodate the petabytes of data being produced by the community.

Metavir, launched in 2011 as an online tool for analysing and visualizing viral taxonomic diversity (Roux et al., 2011), was a major advance for the analytical processing of viral metagenomic datasets. Whilst the initial release focused on single read analyses, the 2014 revision Metavir2 expanded the analysis to assembled viromes and also made significant improvements to enable comparative taxonomic analyses (Roux et al., 2014). However, Metavir2 does not incorporate analysis of viral function and users have limited control over how their data is analyzed. The analysis pipeline and online resource Viral Informatics Resource for Metagenome Exploration (VIROME) was released in 2012 to enable taxonomic, functional and gene richness analyses of viral metagenomes (Wommack et al., 2012). However, the limitation to 250,000 reads has greatly restricted its functionality for large community sequence datasets and the detection of low abundant viruses. The computational framework Viral Metagenome Annotation Pipeline (VMGAP) was also developed in 2011 and enables functional analysis of viral metagenomes (Lorenzi et al., 2011). VMGAP facilitated functional annotation of viral metagenomic datasets by assigning function to open reading frames (ORFs) based on multiple pairwise similarity searches to databases including the non-redundant protein database, Protein Family (PFAM/TIGRFAM) protein domains, the classification of mobile genetic elements (ACLAME) database and environmental protein databases (Lorenzi et al., 2011). This tool can also identify protein domains, signal peptides and Enzyme Commission (EC) assignments before producing a detailed annotation of these results for each input ORF. While VMGAP is undoubtedly a robust methodological framework for annotating viral ORFs, it is designed to annotate individual viral sequences and implements sequence similarity searches against 12 database resources, making it computationally expensive to perform, particularly when multiple viral metagenome samples are being compared.

In order to facilitate rapid in-house analysis of marine holobiont viral metagenome sequence data (using the methods in Weynberg et al., 2014), we have developed and validated a flexible and robust script-based workflow that accepts overlapping paired-end Illumina data [after basic Quality Control (QC) and trimming]. It returns taxonomic annotation for single reads and assembled contigs, as well as gene prediction and functional analysis. HoloVir has been designed for the analysis of DNA viral metagenomics, and its protocols would need to be modified in order to analyse RNA viral metagenomics datasets. The application of HoloVir is demonstrated for two marine invertebrate-associated viral metagenome communities.

## Methods

### Simulated viral metagenomes

To determine the optimal assembly algorithm for viral metagenomes from holobiont samples and evaluate whether nested assembly improves genome reconstruction, two mock viromes were simulated from known viral genomes using art_illumina (Huang et al., 2012) with the following parameters: -p –l 250 –m 450 –s 10. The first simulated dataset contained 5 taxonomically distinct viruses in varying abundance (Table S1) and the second comprised 10 viruses including three closely related Cyanophage species (Table S1). Each mock community contained three million 250 base pair (bp) overlapping paired end Illumina MiSeq reads, representing typical sequencing characteristics for holobiont viral metagenomes (Weynberg et al., 2014). Simulated metagenomics datasets and the original source genomic information is included in the github repository for Holovir (https://github.com/plaffy/HoloVir).

Simulated viral metagenomes were assembled using CLC Genomics Workbench 8.5.1 (https://www.qiagenbioinformatics.com/), Ray Meta (Boisvert et al., 2012), IDBA-UD (Peng et al., 2012) and Trinity (Grabherr et al., 2011) to determine which software produced the most complete assembly, defined as the total proportion of source genomic composition that could be reconstructed. All assemblies were performed using default parameters and Ray Meta incorporated a kmer length of 31 unless otherwise stated. Failure to assemble Cyanophage PSS2 contigs within initial Ray Meta assemblies was resolved by repeating the assembly process incorporating a kmer length of 21. For all assemblies, a minimum contig size of 1000 bp was used in order to increase assembly reliability (Mende et al., 2013). Assembled contigs were aligned to their corresponding reference genomes using the MUMmer bioinformatics software suite (Kurtz et al., 2004). The comparison script run_mummer3 compares contigs to genomes and was used to determine which assembly algorithm produced the highest coverage of the original genomes.

In order to identify the optimal software tool for gene prediction in viral metagenome datasets, gene prediction was performed on both simulated datasets using four different metagenomic gene prediction software tools; FragGeneScan (Rho et al., 2010), MetaGeneAnnotator (Noguchi et al., 2008), Orphelia (Hoff et al., 2009) and MetaGeneMark (Zhu et al., 2010). The original CDS annotations of each genome within the simulated datasets were used to determine the performance and accuracy of each gene prediction method. The total number of predicted genes which were identical or overlapped genomic CDS annotations was determined, as well as the number of annotated genes that are not identified in each gene prediction method, and these results were compared to each other in order to evaluate the performance of each gene prediction algorithm.

### Collection of marine invertebrates for viral metagenomics

To demonstrate the utility of HoloVir in typical marine holobionts, samples from two different invertebrate phyla were processed. Viral extracts were prepared from the Scleractinian coral *Pocillopora damicornis* and the marine Demosponge *Rhopaloeides odorabile*. *P. damicornis* (*n* = 3) were sampled at Trunk Reef (18°20.49′S, 146°49.46′E) in November 2012 and processed as described in Weynberg et al. (2014). Briefly, coral tissue was blasted from the skeleton using an air-gun into 15 ml 0.02 μm filtered (Anotop, Whatman) SM buffer (100 mM NaCl, 8 mM MgSO4.50 mM Tris pH 7.5) in a zip- lock bag. *R. odorabile* (*n* = 3) were collected from Davies Reef (18°50.558′S, 147°37.618′E) in January 2014 and samples were processed by excising 5 cm^3^ pieces of tissue incorporating both pinacoderm and mesohyl layers as described previously (Burja et al., 1998).

### Sample homogenization and cellular disruption

Samples of *P. damicornis* were homogenized and membranes disrupted as previously described (Weynberg et al., 2014). Briefly, blastate from all biological replicates was pooled prior to homogenization at 10,000 rpm for 1 min and centrifugation at 400 g for 5 min. To disrupt the cells, beating with 425–600 μm diameter acid-washed glass beads was performed on the homogenates at 5000 rpm for 5 min. Samples were centrifuged at 14,000 rpm for 1 min before the supernatant was collected for viral fractionation, snap frozen and stored at −80°C until required. Samples of *R. odorabile* were firstly cut into small pieces using the Tupperware Turbo Chef then homogenized in SM buffer for 10 min or until separation of the tissue and skeleton became evident. *R. odorabile* samples were filtered through a 100 μm sieve (Corning Life Sciences), centrifuged at 500 g for 15 min and the supernatant recovered for further processing.

### Cesium chloride fractionation of cellular isolates

In order to fractionate cellular isolates to capture viruses and virus like particles, physical separation using cesium chloride (CsCl) density gradient centrifugation was performed as previously described (Weynberg et al., 2014). The density of resulting fractions was determined gravimetrically and DNA concentrations of each fraction were measured using a Quant-It Picogreen dsDNA high sensitivity assay kit (Invitrogen, Live Technologies). Fractions containing nucleic acids were pooled together prior to buffer exchange (to remove CsCl salts) using Amicon centrifugal spin columns (30 kDa, Millipore) and 0.02 μm filtered SM buffer. The viscosity of the sponge samples necessitated 0.2 μm filtering prior to buffer exchange. All samples were then filtered using 0.2 μm pore size Durapore® (low protein binding) syringe filters to remove any remaining contamination.

### Nucleic acid extraction, amplification, and sequencing

All samples were treated with DNase (Epicentre) and RNase (MoBio) for 30 min at 37°C prior to nucleic acid extraction. RNase treatment and DNA extraction of the viral extract from *P. damicornis* was performed using a MasterPure kit (Epicentre, Illumina) following manufacturer's instructions. Nuclease treatment and DNA extraction of the viral extract from *R. odorabile* was performed using the FastDNA™ SPIN Kit for Soil (MP Biomedicals) following the manufacturer's instructions.

In order to reduce amplification bias encountered with standard Multi-displacement amplification techniques, all DNA samples were amplified using a modified Random Priming-mediated Sequence-Independent Single-Primer Amplification (SISPA) approach as per Weynberg et al. (2014). Final amplified PCR products were cleaned using a MinElute® PCR purification kit. Samples were checked for quantification using a Quant-iT PicoGreen® kit on a NanoDrop 3300 fluorospectrometer, for quality (260:280 ratios), and were visualized on a 0.8% agarose gel to confirm that a size range appropriate for sequencing (~250–500 bp) was present without contamination of smaller fragments. All viral metagenomes were sequenced using Nextera XT MiSeq 300 bp paired-end sequencing (Illumina) at the Ramaciotti Centre, University of New South Wales, Sydney, Australia. The datasets generated from the *P. damicornis* and *R. odorabile* samples were submitted to Genbank Sequence Read archive and are available under the accession numbers SRX503392 and SRS1228599 respectively.

### Sequence analysis of holobiont viral metagenomes

A two-tiered computational approach based on HoloVir was undertaken on each dataset comprising (i) a QC trimmed single read analysis to determine the taxonomic composition of viruses and (ii) a metagenome sequence assembly followed by gene prediction, taxonomic analysis and functional categorization. Single read and assembled data were directly compared to assess whether the assembly protocol was sufficiently robust to identify both abundant and rare viral taxa and determine the overall functional profile of these metaviromes.

### Single read analysis: QC trimming and paired end merging

Raw sequence reads were processed in CLC Genomics Workbench 8.5.1 (CLC Bio, Aarhus, Denmark), adaptor sequences were trimmed and reads were filtered to ensure an average PHRED score of 20 and a minimum sequence size of 100 bp. Paired reads were merged in CLC Genomics Workbench and a final data set containing merged pairs, and unmerged orphan sequences was combined, before a final sequence minimal length cutoff of 200 bp was applied. In order to reduce the computational costs, samples were dereplicated using CD-HIT (Fu et al., 2012) with a sequence identity threshold of 99%. The dereplicated output was used for all subsequent sequence similarity searches in the read-centric analysis.

### Sequence similarity comparisons of single read viral metagenomes

Comparison to the viral RefSeq database (Brister et al., 2015) is the most popular way to identify reads of potential viral origin (Lorenzi et al., 2011; Wommack et al., 2012; Roux et al., 2014), and also forms the basis for assigning metavirome composition within this computational workflow. BLAST sequence similarity searches to viral RefSeq were performed using default parameters (Altschul et al., 1990). However, without a detailed understanding of the level of cellular contamination in the holobiont metavirome datasets, it is difficult to determine how non-viral reads influence the formation of the inferred viral metagenomic community. A primary limiting factor in the analysis of viral metagenomes is the absence of a complete database of virus-specific marker genes. Whilst a reliable bacteriophage marker dataset exists (Kristensen et al., 2013), this does not currently incorporate eukaryotic viruses within the orthologous group associations, and is therefore unable to identify all potential viruses likely to be found within holobiont datasets.

Potential cellular contamination of the viral datasets was determined by performing a sequence similarity search (using BLAST with default parameters) to a cellular marker gene database containing sequences from two reference databases of phylogenetic markers, namely a ribosomal RNA database (SILVA, release 115) (Quast et al., 2013) and an in-house database of universally conserved proteins found in EggNOG 4.0 (Powell et al., 2014) (Clusters of Orthologous Groups that are encoded in at least 99% of all archaea, bacteria and eukaryote genomes). This cellular marker database was extended with bi-directional best hits from all RefSeq genomes that are not included in EggNOG 4.0. The database of cellular markers has been combined with the proteins from virus-specific phage orthologous groups (Kristensen et al., 2013). Taxonomic assignment was determined using MEGAN5 (Huson et al., 2007). MEGAN5 utilized a lowest common ancestor scoring system to assign taxonomy, maintaining a minimum bitscore threshold of 80, a top-percent parameter set at 80 and a minimum support parameter set at one read (cellular and phage marker database) and five reads (viral RefSeq database).

### Gene-centric analysis: assembly, gene prediction and taxon prediction of viral metagenomes

*De novo* assembly of viral metagenomes was performed using CLC Genomics Workbench 8.5.1, with a subsequent filtering step for a minimum of 3 × coverage and a minimum contig length of 1000 bp. Based on results from the mock community analysis, gene prediction was performed for all holobiont datasets using MetaGeneAnnotator. Predicted genes were screened using the same sequence similarity approach as described above for the single read analysis. Taxonomic assignment was performed using MEGAN5 as described above.

### Functional analysis of viral metagenomes

The functional role of predicted genes from the viral assemblies was determined by performing a BLAST sequence similarity search of predicted genes against the UniprotKB/Swiss-Prot functionally annotated database (Suzek et al., 2007; Consortium, 2015). An *e*-value cutoff of 10^−10^ was applied, SwissProt keywords were identified for each best hit and collated for each viral metagenome and for the entire UniprotKB/Swiss-Prot database as a reference. In order to identify broad functional categories of predicted viral genes for each metagenome, predicted genes were also searched (using an *e*-value cutoff of 10^−10^) against the EggNOG 4.5 database (Huerta-Cepas et al., 2015), which includes 2605 protein orthologous groups from 352 viral genomes. The functional categories assigned to the COG of each best hit within EggNOG 4.5 for each predicted gene were counted to summarize broad protein functions (Galperin et al., 2015).

## Results and discussion

### Design and implementation of HoloVir

#### Mock viral metagenomes

The Mock5 dataset contained five viral genomes, representing species that infect prokaryotic and eukaryotic hosts, with a combined metagenome size of 707,422 bp. The Mock10 dataset contained 10 viral genomes of phages and non-phages with a total genome size of 2,358,048 bp. Three closely related Myovirus genomes were included in the Mock10 dataset to examine how each assembler dealt with the differentiation and assembly of closely related species.

#### *De novo* assembly in HoloVir

Assembly statistics (number of contigs, total number of bases in the assembly, N50 value, size of the longest contig and coverage of the original viral genomes) were collated for each of the assembly tools following analysis of the simulated datasets (Tables 1, S2, and S3). For the Mock5 dataset, CLC Genomics Workbench assembled the largest overall contig (179,062 bp), produced a combined contig size most closely reflecting the original metagenome size (689,270 bp) and covered 98% of the original genomes. While the largest contigs produced by Trinity and Ray Meta were comparable in length to CLC Genomics Workbench (177,419 and 179,062 bp respectively), Trinity assembled a total of 960,610 bp which is considerably larger than the original genome size and Ray Meta failed to assemble any contigs originating from the Podoviral Prochlorococcus phage P-SSP7, as well as covering only 76.7% of the original genomes. When the Ray Meta assembly was repeated using a kmer length of 21, the Podoviral Cyanophage PSS2 contigs were assembled. The IDBA-UD assembly produced numerous small contigs (< 1000 bp), a largest contig size of 97,990 bp and covered only 58.7% of the original genomes. Overall performance of the various assemblers was consistent between the Mock5 and Mock10 datasets, with CLC Genomics Workbench covering the highest proportion of the original Mock10 metagenome (96.7%), having the highest number of bases assembled (2,194,206 bp) and producing the largest contig size (733,564 bp).

**Table 1:** **Assemblies of simulated viral metagenomes with and without contig size filtering**.

**Assembly algorithm**	**Ray meta**		**IDBA-UD**		**Trinity**		**CLC genomics workbench**	
	**All contigs**	**Contigs>1 kb**	**All contigs**	**Contigs>1 kb**	**All contigs**	**Contigs>1 kb**	**All contigs**	**Contigs>1 kb**
**Mock dataset**	**Mock5**							
# bases	601,595	585,524	10,281,842	421,252	968,069	960,610	686,987	**669,719**
Total number of contigs	92	50	64,966	25	116	106	45	16
Longest contig (bp)	179,062	179,062	97,990	97,990	177,419	177,419	182,047	**182,047**
N50	15,944	15,944	187	32,637	24,173	14,026	86,038	**102,178**
% of reference genomes covered	76.9	76.0	98.8	58.8	97.9	**97.5**	98.0	**97.6**
**Mock dataset**	**Mock10**							
# bases	2,218,909	2,185,321	9,635,750	2,016,524	3,027,437	2,988,389	2,361,691	**2,218,543**
Total number of contigs	203	73	49,720	95	358	308	326	64
Longest contig (bp)	276,216	276,216	868,737	868,737	130,081	130,081	747,574	**745,626**
N50	129,841	129,841	199	**176,790**	14,000	24,473	131,252	133,117
% of reference genomes covered	88.3	87.4	99.0	59.9	94.7	91.2	98.1	**96.7**

*Assembly statistics are provided for two mock viral metagenomes using four different assemblers, Ray, IDBA-UD, Trinity and CLC Genomics Workbench de novo assembler. For each assembly, statistics are listed for all contigs and for contigs with a minimum size of 1000 bp. The total coverage of the reference genomes was calculated using run_mummer3. Best values for longest contig, N50 and percentage of reference genome covered as well as the total number of bases most closely resembling source genomes size is indicated in bold*.

When investigating the relative performance of each assembler tested in order to differentiate between closely related viruses, Ray Meta and CLC Genomics workbench were able to reconstruct 100% of the original genomes of Prochlorococcus phage P-SMM3 and Cyanophage P-RSM1, while Trinity assembled only 95.5 and 87% respectively. For Prochorococcus phage P-SMM4, which was less prevalent in the simulated community and shared 91% sequence identity to P-SMM3, Ray Meta, CLC Genomics Workbench and Trinity reconstructed 88.2, 78.7, and 57.1% of the original genome respectively. Based on these findings we can conclude that Ray Meta and CLC Genomics Workbench were suited to resolve strain variation in viral metagenomics datasets.

This comparative analysis of simulated viral metagenomes revealed that the commercially available *de novo* assembler within CLC Genomics Workbench performed well for both simple and more complex viral metagenomes, and was hence incorporated into the HoloVir workflow. However, it is important to note that freely available assemblers could be easily substituted if required, allowing for continued flexibility of HoloVir as new assemblers and sequencing platforms are developed. Importantly, while Ray Meta performed well at assembling contigs from closely related viral species, it failed to assemble contigs from the Prochlorococcus phage P-SSP7 using the widely used kmer setting of 31. Although contigs of this virus were successfully assembled with *k* = 21, the optimal kmer values for different biological datasets would be difficult to determine *a priori*. Therefore, assemblies based on different kmer settings should be combined when using Ray Meta. The number of bases assembled into contigs using Trinity was considerably larger than the total size of the reference genomes for both simulated datasets (Table 1), indicating that Trinity tends to assemble multiple variants of contigs. This observation is not unexpected, as Trinity is primarily designed to assemble RNA-seq datasets and is optimized for detecting different splice variants of genes. While coverage remained high in Trinity assemblies (Table 1), the variation in sequences that Trinity is identifying is not present in the Mock5 community indicating that this assembler is overestimating overall community variation.

#### Binning of viral metagenomics datasets

Recent developments in metagenomic sequence binning have revolutionized the way microbial metagenomes are analyzed and greatly improved our ability to close microbial genomes (Brady and Salzberg, 2009; Imelfort et al., 2014; Laczny et al., 2015). However, while the capacity to produce distinct viral sequence bins would undoubtedly improve interpretation of holobiont-derived viral metagenomes, a recent investigation into viral metagenome binning confirmed that coverage-based binning methodologies are not appropriate for amplified viral samples (Smits et al., 2015). Tetranucleotide frequency binning has been used in several metagenomics analyses to identify discrete microbial bins (Swingley et al., 2012; Delmont et al., 2015; Moreira et al., 2015; Ngeow et al., 2015). However, this method requires contigs of at least 5kb for reasonable accuracy (Dick et al., 2009). The majority of our assembled contigs from biological datasets are less than 5 kb in length, limiting the use of this binning strategy at this point in time. Future methodological advances such as the generation of longer sequence reads or the ability to sequence unamplified template are likely to enhance the utility of binning strategies in holobiont-derived viral metagenomes. Consequently, HoloVir does not as yet perform any binning of assembled contigs.

#### Gene prediction within HoloVir

The genomes used to generate the Mock5 and Mock10 simulated datasets contained 875 and 2140 reference genes respectively. Gene prediction was performed on CLC Genomics Workbench assembled Mock5 and Mock10 contigs using the four candidate gene prediction tools. In Mock5 and Mock10 simulated metagenome assemblies, MetaGeneAnnotator produced the greatest percentage of correct predictions with 72 and 80% respectively (Figure S1). MetaGeneMark correctly predicted 70 and 73% of genes in the Mock5 and Mock10 assemblies respectively. FragGeneScan and Orphelia predicted the least number of genes correctly from both simulated dataset assemblies (Figure S1).

This comparative analysis of four different gene prediction tools using simulated data identified MetaGeneAnnotator and MetaGeneMark to both provide accurate gene predictions, identifying more than 70% of all genes, and identifying correct stop codon regions for more than 90% of all genomic coding sequences (Figure S1). MetaGeneAnnotator was initially designed to predict both phage and prokaryotic genes (Noguchi et al., 2008) and has been designed to accommodate overlapping ORFs. Based on the results of our gene prediction analysis, MetaGeneAnnotator gene prediction was incorporated into the HoloVir workflow, however any appropriate gene prediction tool could be incorporated into this workflow as the field progresses.

#### Taxonomic analysis of viral metagenomes in HoloVir

The overwhelming presence of genomic material from lysogenic viruses distributed throughout cellular genomes invariably means that cellular genomic resources are littered with unidentified viral orphans that can significantly hinder identification of viral sequences (Soffer et al., 2014). In addition, databases are biased toward cellular proteins as highlighted by the NCBIs Entrez database, which contains 3.1 million viral proteins compared to 31.6 million eukaryotic proteins and 180.6 million bacterial proteins (NCBI, 2015). For this reason, most viral metagenome studies utilize the exclusively viral RefSeq database (Roux et al., 2014; Soffer et al., 2014; Weynberg et al., 2014), which is not capable of detecting cellular contamination in metavirome data sets. To overcome this limitation, HoloVir uses a cellular marker database to identify potential cellular contamination along with comparisons to phage-specific sequence clusters (Kristensen et al., 2013) to complement viral RefSeq phage assignments. As the HoloVir pipeline was developed to investigate viral assemblages associated with invertebrate holobionts, its marker database also incorporates eukaryotic, bacterial and archaeal gene markers although alternative bacterial marker datasets, such as those generated through PhyloSift (Darling et al., 2014), can also be used for validation. Including a cellular and virus marker database for viral RefSeq validation is essential to ensure that taxonomic assignment parameters are stringent enough to provide accurate composition of viral metagenomes. HoloVir also utilized a two-tiered taxonomic analysis that performs assignments on both single read data and genes predicted from assembled data. This complementary approach can provide confirmation of community assignments, increasing the overall confidence of the analysis.

#### Functional analysis of viral metagenomes in HoloVir

Investigations of microbial metagenomes in previous studies (Anderson et al., 2014; Vázquez-Castellanos et al., 2014) have made functional assignments utilizing existing genomic resources, including Clusters of Orthologous Groups (COG), the SEED database and the Kyoto Encyclopaedia of Genes and Genomes (KEGG) (Anderson et al., 2014). SEED is a framework of subsystem annotations generated from bacterial and archaeal genomes within the FIGfam database and while FIGfam currently includes 1713 viral genomes, this genomic information is yet to be incorporated into the SEED subsystem annotations (Meyer et al., 2009). Phage SEED classifications have been developed as a part of the PhAnToMe phage annotation and analysis project (http://www.phantome.org/). All phage subsystems that have been curated have been included, however only 40 different subsystems have been classified and they are all limited to bacteriophages. KEGG also facilitates functional sequence annotation but while it contains functional information for over 4000 bacteria and eukaryotes, it incorporates no viral genomic data. A recent release of KEGG, termed KOALA (KEGG Orthology and Links Annotation) links existing KEGG orthology assignments with sequences from the RefSeq database, however viral sequences in KOALA remain largely unannotated due to the absence of viral genomic information used to develop the KEGG orthology system (Kanehisa et al., 2015). COG uses complete microbial genomes and orthology based approaches to assign functions using specific protein assignments as well as broad functional classifications (Galperin et al., 2015). A recently developed functional ontology (FOAM) assigns gene functions relevant to environmental microorganisms based on Hidden Markov Models (Prestat et al., 2014). An extension to typical viral functions (“virus structure,” “virus replication” and “virus-host interaction”) has not yet been presented but would be extremely valuable for functional analysis of viral metagenomes. While all the mentioned resources have been invaluable to microbial metagenomics, they are designed to describe cellular functionality (Meyer et al., 2008), hence have limited utility for functional characterization of viral metagenomes.

The Gene Ontology (GO) database incorporates curated functional assignments of protein sequences. Based on sequence data from model organisms, a total of 4267 viral proteins with GO functional annotations are incorporated into the database, although these viral sequences are almost exclusively human pathogens or viruses related to agricultural species and do not cover a wide range of viral taxa. The Swiss-Prot component of the UniprotKB database contains 550,116 manually curated proteins including 16,605 viral sequences comprised of 9228 dsDNA, 4391 single stranded RNA (ssRNA), 1404 retro-transcribing, 889 double stranded RNA (dsRNA) and 612 ssDNA viral sequences. The UniprotKB/Swiss-Prot sequences also contain keyword assignments that facilitate direct functional comparisons between individual viral metagenomes. Within the HoloVir workflow we have therefore incorporated a two-step functional characterization comprising broad classification of COG functions (informative for identifying viral accessory genes present in metavirome communities) and a more targeted analysis of enriched Swiss-Prot keywords.

**Figure 1:**
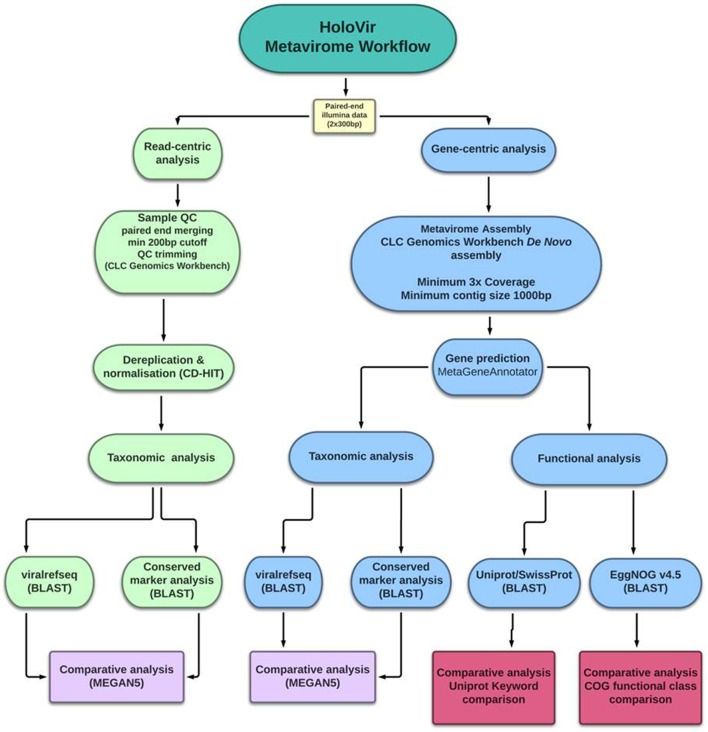
**Graphical overview of HoloVir, the computational workflow for predicting taxonomic composition and gene functions from invertebrate-associated metaviromes**.

Analysis of simulated viral metagenomes has facilitated identification of the optimal assembly and gene prediction strategy for viral metagenomes and review of available genomic resources has further defined the optimal workflow for functional assignment and characterization. HoloVir utilizes the *de novo* assembler in CLC genomics workbench to produce viral contigs, then predicts viral gene sequences using MetaGeneAnnotator. This combination of methods is sensitive enough to assemble both simple and more complex viral communities, and can account for viral microdiversity in the production of viral contigs. HoloVir utilizes pairwise sequence comparisons to the viral RefSeq database in order to assign taxonomy to both single reads and predicted genes. A cellular and phage marker dataset was also used to confirm phage taxonomic assignment and identify potential cellular contamination. Finally, HoloVir performs broad community functional assignment using EggNOG 4.5 and UniprotKB/SwissProt comparisons to infer gene functions (Figure 1). Although initially designed for analysis of holobiont-associated viral metagenomics analysis, its use could also be broadened into any viral metagenomic studies. HoloVir is implemented as a collection of Linux shell scripts and is freely available on github (https://github.com/plaffy/HoloVir).

### Application of HoloVir using biological samples

#### Analysis of holobiont viral metagenomes: sequence preparation and QC

Sequencing of *P. damicornis* and *R. odorabile* viromes produced 9,348,233 and 11,893,822 raw reads respectively. After QC, size filtering, and merging paired ends, a total of 2,646,987 high quality reads (200–488 bp) remained for *P. damicornis* and 8,593,363 (200–587 bp) remained for *R. odorabile*. In order to reduce computational requirements for processing, single reads were clustered at 99% sequence identity and dereplicated, yielding 329,456 reads for *P. damicornis* and 499,282 reads for *R. odorabile* (summarized in Table S4).

#### Assembly of holobiont viral metagenomes and gene prediction

*De novo* assembly of viral metagenomes derived from *P. damicornis* and *R. odorabile* using CLC Genomics Workbench produced 10,749 and 2739 contigs respectively (Table S4). The longest contigs produced for *P. damicornis* and *R. odorabile* were 66,342 bp and 16,812 bp respectively, and corresponding N50 values of 1682 bp and 1776 bp were observed. Following gene prediction using MetaGeneAnnotator, a total of 31,010 *P. damicornis* and 8416 *R. odorabile* genes were predicted. These predicted genes were used for the gene-centric component of HoloVir.

#### Taxonomic assignment of holobiont viral metagenomes

**Figure 2:**
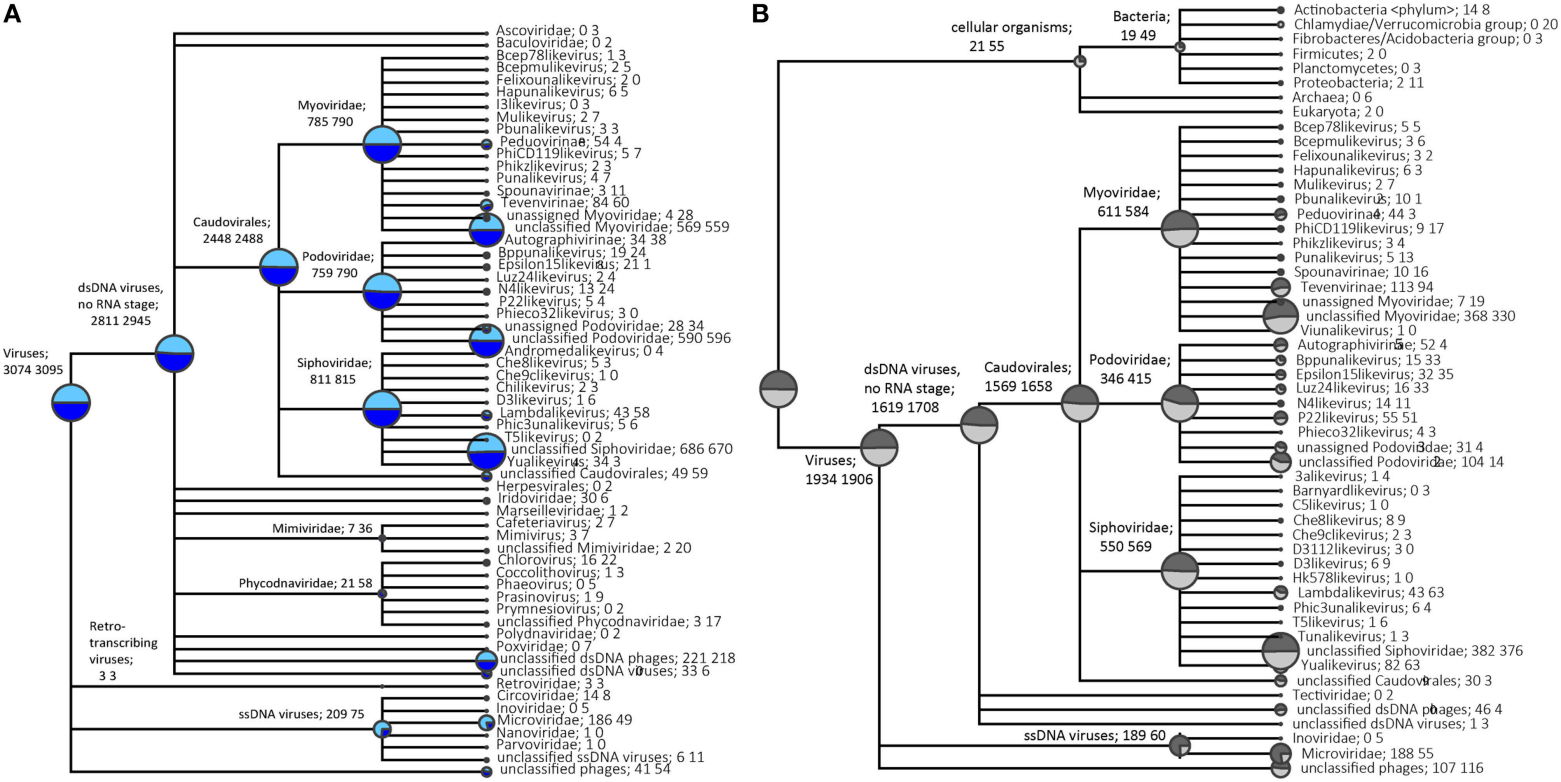
**Taxonomic overview of the ***P. damicornis*** viral metagenome**. Normalized taxonomic assignments of the metavirome data sets using NCBI's viral RefSeq database as BLAST searches from single read analysis (in light blue) and predicted genes from assembled data (in dark blue) are displayed in **(A)**. The size of the colored circle is indicative of the relative abundance of reads in the metavirome being assigned to each specific taxonomic level (square root scaled). Normalized taxonomic assignments of the metavirome data sets against phage-specific orthologous group (POG) and cellular marker database as BLAST searches from single read analysis (in light gray) and predicted genes from assembled data (in dark gray) are displayed in **(B)**. The MEGAN5 last common ancestor classification was used to assign all taxonomy. Data sets were normalized against the total number of significant assignments using a minimum bitscore threshold of 80, with taxonomic assignments being made based on 80% consensus of the best BLAST matches.

Taxonomic assignment of single-reads and predicted genes from the assemblies was performed following BLAST searches against the NCBI viral RefSeq database (Figure 2A, Figure S2A) and custom phage-specific and cellular marker databases (Figure 2B, Figure S2B). For the *P. damicornis* single read and predicted gene data sets, 19,654 and 1782 sequences respectively were assigned taxonomy using viral RefSeq. 11,914 and 1585 respectively matched to the phage-specific marker database and 143 and 13 respectively matched to the cellular marker database (Figures S3, S4). For the *R. odorabile* dereplicated single read and predicted gene data sets, 19,618 and 689 sequences respectively were assigned taxonomy using viral RefSeq, 16,719 and 623 respectively matched to the phage-specific marker database and 191 and 1 respectively matched to the cellular marker database (Figures S5, S6).

A normalized comparison between assigned viral RefSeq matches from read-centric and gene-centric data for *P. damicornis* was performed (Figure 2A), as well as a similar normalized comparison for the phage-specific and cellular markers (Figure 2B). In order to demonstrate differences in taxonomic assignments between the read- and gene-centric approaches, we provide a detailed report for *P. damicornis* (results from the *R. odorabile* comparisons can be found in Figure S2). Using the viral RefSeq assignments, 3074 single reads and 3095 predicted genes were attributed to dsDNA viruses, with 91.4% of assigned single read and 95.2% of predicted genes annotated as bacteriophage in the Order Caudovirales (Figure 2A). A total of 6.8% single reads and 2.4% predicted genes were assigned to ssDNA viruses, three retro-transcribing virus matches were identified from both single read and predicted genes, and 1.3% unclassified phage assignments were made for single reads, and 1.7% from predicted genes. Taxonomic annotation using viral RefSeq identified Caudovirales as the dominant group for dsDNA viral assignments. This was confirmed by the phage-specific marker assignments which assigned 15,698 single reads and 1658 predicted genes to Caudovirales (Figures S3B, S5B). In a normalized comparison of phage-specific marker assignments (Figure 2B), almost three times as many ssDNA reads were assigned, compared to the predicted genes for both viral RefSeq and phage-specific marker analyses. This was largely due to an abundance of Microviridae assignments that were not well represented in the assembled predicted genes (Figure 2). In addition, subfamily-assignments, such as the Felixouna-like virus, were present in the single read analysis but absent in the predicted gene analysis.

Several non-phage assignments could also be made from viral RefSeq analysis of single read and predicted gene datasets, with Mimiviridae, Phycodnaviridae, Poxviridae, Polydnaviridae and Retroviridae assignments all observed. With the exception of Retroviridae, a greater number of assignments were provided by predicted gene analysis compared to single read analysis and Polydnaviridae and Poxviridae were only assigned in the predicted gene data set (Figure 2A). This increased detection in predicted gene data is likely due to the assembly process, which facilitates the production of longer sequences, increasing the likelihood that significant BLAST results will be returned.

In assessing the distribution of marker matches on assembled contigs, a total of 1411 contigs returned a single POG marker match and 235 contigs returned multiple matches. For the contigs with multiple matches, 233 returned non-contradictory taxonomic assignments and two returned contradictory taxonomic assignments (not from the same viral group). A total of 388 *R. odorabile* contigs returned a single POG marker match and 96 contigs returned multiple matches, although all of these were non-contradictory taxonomic assignments. Non-contradictory POG marker assignments provide further support for taxonomic classifications of predicted genes.

#### Comparison of metavirome composition between samples

**Figure 3:**
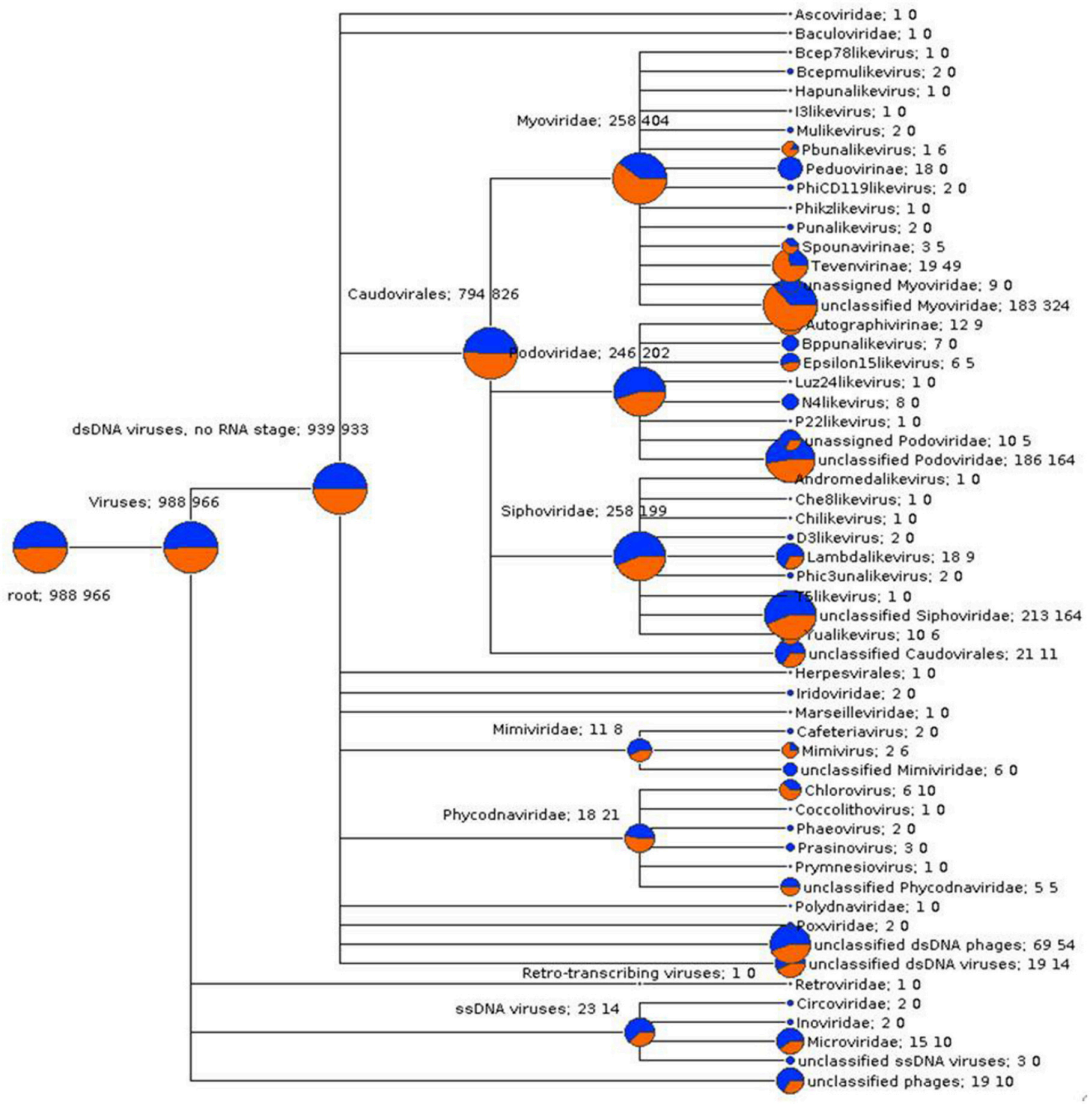
**A gene-centric comparison of the taxonomic composition of viral metagenomes from ***P. damicornis*** (blue) and ***R. odorabile*** (orange)**. Output is based on BLAST analysis of MetaGeneAnnotator predicted genes from assembled metaviromes, with taxonomy of genes assigned using the MEGAN5 last common ancestor classification, a minimum bitscore threshold of 80 and assignments being made using a minimum 80% consensus of the best BLAST matches. The size of the colored circle is indicative of the relative abundance of reads in the metavirome being assigned to each specific taxonomic level (square root scaled). Specific counts of genes that can be assigned to specific taxa are listed to the right of the taxa name (*P. damicornis* on the left, *R. odorabile* on the right).

In order to compare viral community composition across holobiont species, a normalized comparison of viral RefSeq assignments was performed on single reads (Figure S7) and predicted genes (Figure 3) from *P. damicornis* and *R. odorabile* using MEGAN5. Following normalization between datasets, the majority of viral assignments of the predicted genes were to dsDNA viruses (95 and 96.5% for *P. damicornis* and *R. odorabile* respectively), with only a small proportion of assignments made to ssDNA viruses (2.3 and 1.4% for *P. damicornis*, and *R. odorabile* respectively) and retro-transcribing viruses (0.1% for *P. damicornis* and none for *R. odorabile*) (Figure 3). Bacteriophage in the order *Caudovirales* dominated all viral assignments in both datasets, but the distribution of *Caudovirales* families differed between holobiont taxa, with a greater number of *Siphoviridae* and *Podoviridae* assignments in *P. damicornis* and a greater number of *Myoviridae* assignments in *R. odorabile*. Similarly, variation between holobionts were also observed for ssDNA and retro-transcribing viruses, with *Circoviridae, Inoviridae, Poxviridae, Polydnaviridae* and *Retroviridae* only occurring in *P. damicornis* (Figure 3).

#### Assessing cellular contamination of viral datasets based on universal marker genes

The cellular marker database was used to identify possible contaminating cellular sequences. A total of 21 single-read and 55 predicted gene assignments were made to bacterial marker genes in our normalized comparison (Figure 2B). Closer inspection of the specific marker assignments from the predicted genes identified five matches to a DNA-directed RNA polymerase and 24 matches to tRNA-synthetase genes, as well as 10 ribosomal protein genes, four translation elongation factor genes, nine thiol-disulfide isomerase genes and one EMAP domain protein. RNA polymerase genes are often found in DNA viruses as they play a key role in viral genome replication and transcription (Sonntag and Darai, 1995), and tRNA-synthetases have been reported in genomes of several large viruses (Abergel et al., 2007; Yutin and Koonin, 2012; Yutin et al., 2014). Similarly, translational elongation factors have been identified as essential cofactors of RNA-dependant RNA polymerases in RNA bacteriophages (Li et al., 2013). We therefore conclude that most of the hits to the cellular marker proteins are actually viral proteins, from so far unknown lineages and thus having slightly higher sequence similarity to cellular rather than viral reference sequences.

#### Functional assignment of predicted viral genes

**Figure 4:**
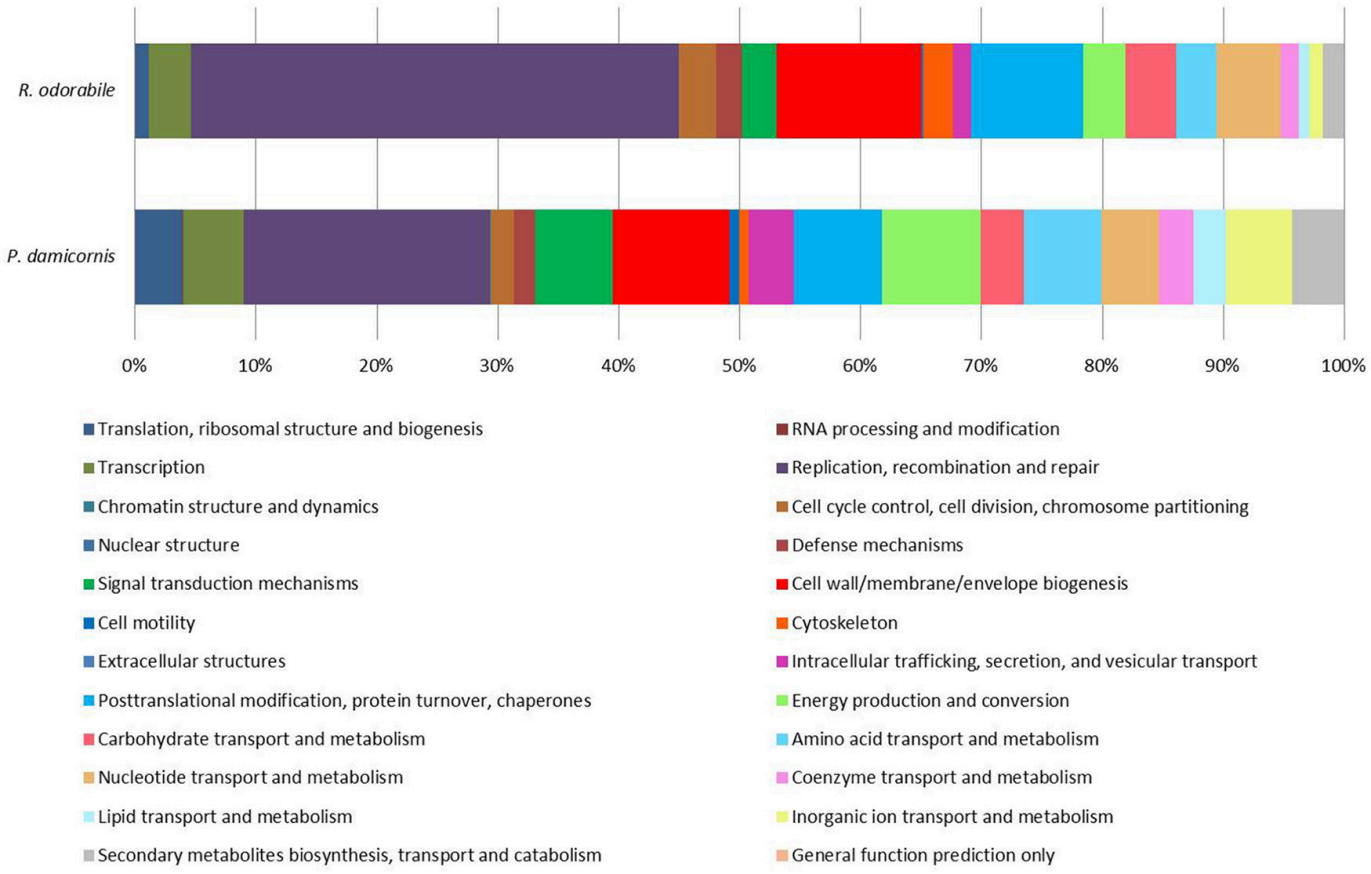
**Functional assignment of predicted viral genes based on COG functional category classification**. A total of 6560 *P. damicornis* and 1041 *R. odorabile* COG functional category classifications were made based on BLAST comparisons to the EggNOG 4.5 database. Of these classifications, 51.6% of *P. damicornis* genes and 56.4% of *R. odorabile* genes were assigned “Function unknown.” The relative proportion of each known COG functional category for genes predicted from viral metagenome of *P. damicornis* and *R. odorabile* are shown.

Predicted genes were assigned to COG functional categories within the EggNOG 4.5 database. A total of 6560 COG functional categories were assigned for *P. damicornis* and 1041 for *R. odorabile*, of which 3172 and 454 respectively were categorized as “function unknown” (Figure 4). In addition, Swissprot keywords were assigned to predicted genes in the UniprotKB/Swiss-Prot database and using the overall frequency of these keyword assignments, 159 and 110 functions were found to be enriched in *P. damicornis* and *R. odorabile* respectively and a further 135 and 118 functions were found to be under-represented in *P. damicornis* and *R. odorabile* respectively. The top 20 enriched Swiss-Prot keywords in both datasets are listed in Table 2 and the total keyword assignments are provided in Table S5. Keywords most enriched in the holobiont datasets included viral functions involved in infection, replication and structural assembly (Table 2).

**Table 2:** **Keyword assignments were identified for the best significant UniprotKB/Swiss-Prot BLAST match for each predicted gene**.

**Swissprot keywords**	***P. damicornis***	***R. odorabile***
Degradation of host chromosome by virus	126.3	548.6
Evasion of bacteria-mediated translation shutoff by virus	202	365.7
Degradation of host lipopolysaccharides during virus entry	101	365.7
Bacterial host gene expression shutoff by virus	84.2	365.7
Viral DNA replication	82.6	359.1
Viral long flexible tail ejection system	256.4	337.6
Viral short tail ejection system	314.2	243.8
Latency-replication switch	N/A	274.3
Viral genome ejection through host cell envelope	156.2	205.7
Viral latency	N/A	182.9
Viral genome excision	15.2	164.6
Viral contractile tail ejection system	67.3	162.5
Viral genome packaging	103.7	151.2
Restriction system	23.2	130.3
Viral capsid assembly	125.5	125.4
Viral baseplate protein	48.9	106.2
Viral tail assembly	83.9	44.5
DNA invertase	79.7	57.7
Viral tail protein	43.9	71.8
Viral tail fiber protein	60.6	62.7

*Enriched functions were determined by comparison of the relative keyword frequency in each dataset with the frequency in the UniprotKB/Swiss-Prot database. The fold enrichments of the 20 most enriched functions are displayed for each host species*.

## Conclusion

HoloVir is a robust and flexible analysis workflow for investigating the taxonomic composition and gene functions of viral communities associated with invertebrate holobionts across environments, species or experimental treatments. Key computational methods were validated using simulated datasets and accordingly implemented in HoloVir. The utility of the workflow was demonstrated on two distinct holobiont-associated viral metagenomes. The workflow has been shown to be flexible enough to accommodate taxonomically diverse hosts, yet specific enough to identify differences within the associated viral assemblages. Visualization of output data can be specifically tailored to complement the scientific focus. For instance, here we visualized taxonomic composition using MEGAN5 and functional composition using COG functional category classifications and enrichment/depletion analysis of Swiss-Prot keywords. However, heatmaps or pathway-level visualization tools that identify key differences in function across viral metagenomes may also be appropriate for larger sample sets. HoloVir provides a valuable tool for investigating viruses associated with invertebrate holobionts and is freely available upon request.

The open source code for HoloVir, and the mock community datasets analyzed in this manuscript are publically available at https://github.com/plaffy/HoloVir.

## Author contributions

PL, NW, TR, KW, MV, EW, EB, DT provided substantial contribution to the conception and design of the work. PL, EW, KW, EB acquired analyzed and interpreted the work. PL, NW, and EW constructed the manuscript. PL, NW, TR, KW, MV, EW, EB, DT were involved in drafting and revising the work and provided final approval of the manuscript for publication. PL, NW, TR, KW, MV, EW, EB, DT agree to be accountable for all aspects of the work.

### Conflict of interest statement

The authors declare that the research was conducted in the absence of any commercial or financial relationships that could be construed as a potential conflict of interest.
