# Supplementary material for: HoloVir: A Workflow for Investigating the Diversity and Function of Viruses in Invertebrate Holobionts
Source: Front Microbiol. 2016 Jun 9;7:822. doi: 10.3389/fmicb.2016.00822 (PMC4899465; doi:10.3389/fmicb.2016.00822)
Supplement: Supplementary file 1 [file DataSheet1.PDF]

Methods: *Hierarchical assembly of simulated viral metagenomes*

A two-step hierarchical assembly was applied to test if the reconstruction of genomes could be improved. In this approach, simulated datasets were subsampled at 50%, 20%, 10%, 5%, 2% or 1% of the original reads and each subsampled dataset was assembled as described above. The original simulated reads were subsequently mapped onto the first round contigs using BWA-SW (Li and Durbin, 2010), and non-mapping reads were obtained using SAMtools (Li et al., 2009) for a second round of assembly using the same parameters. Contigs produced from both rounds of assembly were combined and their coverage of the original viral genomes was performed as described above to determine whether single or hierarchical assembly produced better results.

Results and Discussion: *Hierarchical assembly of viral metagenomics datasets*

A hierarchical assembly was performed on Mock5 and Mock10 datasets using Ray Meta, CLC Genomics Workbench and Trinity. In most instances, hierarchical assembly of the Mock5 dataset produced contigs with lower overall genome coverage than single round assembly (Table S2). The rarest genome in the Mock5 community (Herpesvirus Suid1) consistently lost genome coverage as the percentage of the original first-round data decreased by subsampling. The maximum genome coverage of the Phycodnaviral *Ectocarpus siliculosus* virus 1 (which made up 10% of the Mock5 dataset (Table S1)) varied across the hierarchical assemblies and where hierarchical coverage was higher than a single round assembly, this difference was marginal. In the Mock10 dataset, hierarchical assembly with Trinity increased genome coverage in some cases but this was not observed with Ray Meta. The overall genome coverage of the rarest viruses for CLC Genomics Workbench contigs was reduced in comparison to the single round assembly (Table S3).

Analysis of contigs following hierarchical assembly of simulated viral metagenomes revealed that single round assembly performs better than a two tiered approach for all assemblers tested. While the hierarchical strategy resulted in slightly greater genome coverage of some viruses, the optimal level of subsampling varied between assemblers and between the simulated communities. Importantly, the coverage of the low abundant viruses within the simulated datasets was greatly reduced in some hierarchical assemblies. Without reference sequences available to calculate overall coverage within biological samples, using a hierarchical assembly strategy may reduce the coverage of rare viral taxa. Because of these findings, HoloVir performs a single round of assembly using the CLC Genomics Workbench de novo Assembler, and does not incorporate a hierarchical assembly process.

Table S1. Composition of mock viral metagenome datasets. Prochlorococcus phage P-SSM3 and Cyanophage P-RSM1 share 77% sequence identity, and Prochlorococcus phage P-SSM3 and Prochlorococcus phage P-SMM4 share 91% sequence identity.

					Mock5		Mock10	
	**Taxonomic family**	**Accession number**	**Genome size (bp)**	**Annotated gene count**	**% reads of total mock community**	**Number of reads per species in final community**	**% reads of total mock community**	**Number of reads per species in final community**
**Prochlorococcus phage P-SSM3**	Myoviridae	NC_021559.1	179063	214	40	1200000	15	450000
**Cyanophage P-RSM1**	Myoviridae	NC_021071.1	177211	212	-	-	10	300000
**Prochlorococcus phage P-SSM4**	Myoviridae	NC_006884.2	178249	221	20	600000	5	150000
**Cyanophage PSS2**	Siphoviridae	NC_013021.1	107530	131	29.8	894000	15	450000
**Prochlorococcus phage P-SSP7**	Podoviridae	NC_006882.2	179063	58	-	-	15	450000
**Ectocarpus siliculosus virus 1**	Phycodnaviridae	NC_002687.1	335593	240	10	300000	10	300000
**Acanthamoeba polyphaga mimivirus**	Mimiviridae	NC_014649.1	1181549	979	-	-	15	450000
**Marine gokushovirus isolate GOM**	Microviridae	NC_022790.1	4129	6	-	-	10	300000
**Enterobacteria phage alpha3**	Microviridae	NC_001330.1	6087	10	-	-	4.8	144000
**Suid herpesvirus 1**	Herpesviridae	NC_006151.1	143461	69	0.2	6000	0.2	6000

Table S2. Individual and average genome coverage of assembled contigs from hierarchical two-step assembly of the Mock5 dataset. Ray Meta, CLC Genomics Workbench and Trinity were used for all assemblies. The degree of sub-sampling for the first step is given in the first column. Coverage is listed as a percentage of the original genome that is represented within the metagenome assembly.

	**Prochlorococcus phage P-SSM3**			**Prochlorococcus phage P-SSP7**			**Marine gokushovirus isolate GOM**			**Ectocarpus siliculosus virus 1**			**Suid herpesvirus 1**			**Total average coverage**		
	Ray	CLC	trinity	Ray	CLC	trinity	Ray	CLC	trinity	Ray	CLC	trinity	Ray	CLC	Trinity	Ray	CLC	Trinity
**Mock5 assembly (%)**	100.0	100.0	99.9	0.1	100.0	100.0	100.0	98.5	100.0	83.4	92.1	90.8	96.4	97.5	96.7	76.0	97.6	97.5
**Mock5 50% hierarchical assembly**	100.0	100.0	100.0	0.1	100.0	100.0	100.0	100.0	100.0	84.3	91.0	90.9	79.0	97.2	95.9	72.7	97.6	97.4
**Mock5 20% hierarchical assembly**	100.0	100.0	100.0	0.1	100.0	100.0	100.0	100.0	100.0	85.3	89.7	90.8	11.5	90.6	87.3	59.4	96.1	95.6
**Mock5 10% hierarchical assembly**	100.0	99.9	100.0	0.1	100.0	100.0	100.0	100.0	100.0	86.3	89.7	91.2	0.1	51.0	53.4	57.3	88.1	88.9
**Mock5 05% hierarchical assembly**	100.0	99.9	100.0	0.1	100.0	100.0	100.0	100.0	100.0	84.8	90.1	91.2	0.1	14.5	12.7	57.0	80.9	80.8
**Mock5 02% hierarchical assembly**	100.0	100.0	100.0	0.1	100.0	100.0	100.0	100.0	100.0	59.6	88.9	90.1	0.1	2.8	2.3	52.0	78.3	78.5
**Mock5 01% hierarchical assembly**	100.0	99.4	100.0	0.1	100.0	100.0	100.0	100.0	100.0	5.9	75.5	81.4	0.0	0.1	0.1	41.2	75.0	76.3

Table S3. Original genome coverage of assembled contigs from hierarchical two-step assembly of the Mock10 dataset. Ray Meta, CLC Genomics Workbench and Trinity were used for all assemblies. The degree of sub-sampling for the first step is given in the first column.

	Prochlorococcus phage P-SSM3			Cyanophage P-RSM1			Prochlorococcus phage P-SSM4			Cyanophage PSS2			Prochlorococcus phage P-SSP7		
	Ray	CLC	trinity	Ray	CLC	trinity	Ray	CLC	trinity	Ray	CLC	trinity	Ray	CLC	trinity
**Mock10 assembly (%)**	100.0	100.0	95.5	100.0	100.0	87.0	88.2	78.7	57.1	98.6	99.4	99.4	0.1	100.0	100.0
**Mock10 50% hierarchical assembly**	100.0	100.0	98.5	100.0	100.0	86.8	64.7	66.7	58.0	98.6	99.4	98.9	0.1	100.0	100.0
**Mock10 20% hierarchical assembly**	100.0	100.0	99.9	100.0	100.0	86.9	89.3	61.3	59.1	98.5	99.4	99.4	0.1	100.0	100.0
**Mock10 10% hierarchical assembly**	100.0	100.0	99.0	100.0	100.0	86.0	60.5	59.2	58.7	98.5	99.4	99.4	0.1	100.0	100.0
**Mock10 05% hierarchical assembly**	100.0	100.0	100.0	100.0	99.5	84.4	59.2	59.3	60.7	98.5	99.4	99.4	0.1	100.0	100.0
**Mock10 02% hierarchical assembly**	100.0	100.0	100.0	99.5	100.0	83.9	59.2	59.8	62.1	98.9	99.3	99.3	0.1	100.0	100.0
**Mock10 01% hierarchical assembly**	100.0	100.0	99.1	18.7	99.0	82.4	60.4	60.4	62.5	83.0	99.3	99.3	0.1	100.0	100.0

	Ectocarpus siliculosus virus 1			Acanthamoeba polyphaga mimivirus			Marine gokushovirus isolate GOM			Enterobacteria phage alpha3			Suid herpesvirus 1			Total average coverage		
	Ray	CLC	trinity	Ray	CLC	trinity	Ray	CLC	trinity	Ray	CLC	trinity	Ray	CLC	trinity	Ray	CLC	trinity
**Mock10 assembly (%)**	91.7	91.0	88.2	99.9	99.8	96.1	100.0	100.0	100.0	100.0	100.0	100.0	95.4	98.1	89.0	87.4	96.7	91.2
**Mock10 50% hierarchical assembly**	92.1	89.3	87.3	99.8	99.7	96.3	100.0	100.0	100.0	100.0	100.0	100.0	72.0	97.7	89.9	82.7	95.3	91.6
**Mock10 20% hierarchical assembly**	91.6	89.9	87.8	99.8	99.7	95.9	100.0	100.0	100.0	100.0	100.0	100.0	8.5	84.3	77.0	78.8	93.5	90.6
**Mock10 10% hierarchical assembly**	90.9	89.3	87.3	83.1	99.5	95.7	100.0	100.0	100.0	100.0	100.0	100.0	26.4	30.6	41.3	76.0	87.8	86.7
**Mock10 05% hierarchical assembly**	48.3	87.8	87.7	5.4	92.0	91.4	100.0	100.0	100.0	100.0	100.0	100.0	64.9	32.0	64.6	67.6	87.0	88.8
**Mock10 02% hierarchical assembly**	2.0	69.4	61.9	23.8	31.3	41.2	100.0	100.0	100.0	99.9	99.9	100.0	89.5	71.7	92.2	67.3	83.1	84.1
**Mock10 01% hierarchical assembly**	31.9	25.8	36.7	70.9	34.4	70.6	99.8	99.9	99.9	99.3	99.7	99.7	95.4	90.2	96.4	66.0	80.9	84.6

Table S4.Raw sequence information, contig assembly statistics and gene prediction summary of *P. damicornis* and *R. odorabil*e viral metagenome data.

**Host species**	**Raw read count**	**QC treated read count**	**Dereplicated read count**	**Total contig count (bp)**	**Contig N50 (bp)**	**Longest contig (bp)**	**Total number MGA predicted genes**	**Total number of predicted genes assigned functions against UniprotKB/Swiss-Prot**	**Total number of predicted genes assigned functional group using EggNOG 4.5**
P. damicornis	9,348,233	2,646,987	329,456	10,749	1,682	66,342	31,010	3,718	8,720
R. odorabile	11,893,822	8,593,363	499,282	2,739	1,776	16,812	8,416	513	1,396

Table S5. Keyword assignments were identified for all UniprotKB/Swiss-Prot BLAST matches for viral metagenome predicted genes. Enriched functions were determined by comparison of the relative keyword frequency in each dataset with the frequency in the UniprotKB/Swiss-Prot database. The fold enrichment for each species is displayed.

Uniprot Keyword	*P.damicornis*	*R. odorabile*
Degradation of host chromosome by virus	126.3	548.6
Viral long flexible tail ejection system	256.4	337.6
Evasion of bacteria-mediated translation shutoff by virus	202	365.7
Viral short tail ejection system	314.2	243.8
Degradation of host lipopolysaccharides during virus entry	101	365.7
Bacterial host gene expression shutoff by virus	84.2	365.7
Viral DNA replication	82.6	359.1
Viral genome ejection through host cell envelope	156.2	205.7
Latency-replication switch	-	274.3
Viral genome packaging	103.7	151.2
Viral capsid assembly	125.5	125.4
Viral contractile tail ejection system	67.3	162.5
Viral latency	-	182.9
Viral genome excision	15.2	164.6
Viral baseplate protein	48.9	106.2
Restriction system	23.2	130.3
DNA invertase	79.7	57.7
Viral tail assembly	83.9	44.5
Viral tail fiber protein	60.6	62.7
Viral tail protein	43.9	71.8
Virus exit from host cell	39.8	49.2
Degradation of host cell envelope components during virus entry	18.9	45.7
DNA-directed DNA polymerase	10.7	43
Mucopolysaccharidosis	50.5	-
Whooping cough	50.5	-
Covalent protein-DNA linkage	34.1	13.7
Viral receptor tropism switching	45.5	-
T=1 icosahedral capsid protein	32.8	9.1
Late protein	20.6	19.7
Primosome	17	23.1
Teichoic acid biosynthesis	4.7	34.3
Metachromatic leukodystrophy	37.9	-
Host cell inner membrane	-	36.6
Protein splicing	13.4	21.5
Exopolysaccharide synthesis	15.5	18.7
Host cell lysis by virus	17.6	15.9
Transposable element	10.2	22
Bacteriolytic enzyme	15.9	15.7
Restriction-modification system evasion by virus	30.3	-
Chromate resistance	30.3	-
Antibiotic biosynthesis	20.5	9.6
RNA-directed DNA polymerase	12.9	16.7
Viral attachment to host adhesion receptor	9.6	17.4
Viral penetration into host cytoplasm	11.5	15.1
Ascorbate biosynthesis	3.2	23.3
Transposition	10.8	14.9
T=7 icosahedral capsid protein	24.2	-
Cobalamin	14.6	9.6
Germination	11.7	12.1
Aicardi-Goutieres syndrome	21.6	-
DNA integration	9.2	12.4
DNA replication	5.4	15.1
Capsid inner membrane protein	20.2	-
Viral genome integration	14.4	5.8
Peptidoglycan-anchor	-	19.4
Capsid protein	9.3	10.1
Exonuclease	5.2	14.1
Citrate utilization	18.9	-
Oxylipin biosynthesis	3.9	14.2
Galactose metabolism	2.1	15.3
Virus entry into host cell	7.7	9.4
Starch biosynthesis	-	16.1

T=13 icosahedral capsid protein	3.1	11.2
PQQ	14.2	-
Amyloplast	-	13.9
Bromodomain	-	13.8
Copper transport	5.4	7.9
Cadmium resistance	13	-
Sigma factor	4.9	8
Kelch repeat	-	12.6
Glutamate biosynthesis	12.6	-
Polysaccharide transport	12.2	-
Capsule biogenesis/degradation	12.2	-
Dynein	-	11.9
Conjugation	11.9	-
Virion	4.8	6.4
Bacteriocin biosynthesis	10.8	-
Plasmid	5.5	5.1
Helicase	3.6	6.9
Phosphopantetheine	10	-
Isoleucine biosynthesis	10	-
Two-component regulatory system	9.7	-
TonB box	9.5	-
Nuclease	3.4	6
Lipopolysaccharide biosynthesis	3.2	6.1
Fertilization	3.3	6
Degradation of host peptidoglycans during virus entry	8.9	-
Melatonin biosynthesis	8.9	-
DNA synthesis	5.4	3.5
Viral attachment to host entry receptor	8.7	-
TPR repeat	4	4.6
Photoreceptor protein	8.3	-
Nucleotidyltransferase	2.3	6
Host cytoplasm	3.5	4.8
cAMP biosynthesis	8.1	-
Intron homing	8.1	-
Mercury	7.6	-
Calmodulin-binding	-	7.5
Chromosome partition	2.5	4.9
Early protein	2.7	4.7
DNA recombination	3.1	4.2
Trimethoprim resistance	7.2	-
Glycolate pathway	7.2	-
ERV	7.1	-
Nodulation	3.1	3.8
Mercuric resistance	6.7	-
PHB biosynthesis	6.6	-
Competence	6.5	-
Herbicide resistance	2.3	4.2
Peptide transport	2.6	3.8
WD repeat	-	6.2
Alginate biosynthesis	6.2	-
Leukodystrophy	6.1	-
Maltose metabolism	6.1	-
Flowering	-	6
3D-structure	2.7	3.3
Endonuclease	3	2.8
Cilium biogenesis/degradation	-	5.7
Copper	2.4	3.2
Methotrexate resistance	5.4	-
DNA-binding	2.1	3.3
Nitrate assimilation	5.3	-
Bacterial flagellum protein export	5.2	-
Sulfate transport	5.2	-
Branched-chain amino acid catabolism	5.2	-
Complement activation lectin pathway	5.2	-
Multifunctional enzyme	3.2	2
Fimbrium biogenesis	5.1	-
Inositol biosynthesis	5.1	-
Aspartyl protease	-	4.7
Host gene expression shutoff by virus	-	4.6
Transcription antitermination	4.4	-
Proline metabolism	4.3	-

c-di-GMP	4.3	-
Nucleotide biosynthesis	2.2	2.1
Dipeptidase	-	4.2
Iron transport	4.2	-
Primary ciliary dyskinesia	4.2	-
Lithium	4.2	-
Cadmium	3.9	-
CBS domain	3.8	-
Plasmid copy control	3.8	-
Molybdenum	3.8	-
Ichthyosis	3.7	-
Phosphotransferase system	3.6	-
Leucine-rich repeat	0.4	3.2
Cell wall	-	3.5
Threonine biosynthesis	3.5	-
Serine biosynthesis	-	3.4
Serine esterase	-	3.4
Aminotransferase	-	3.4
Biotin	3.4	-
Ammonia transport	3.3	-
Gluconate utilization	3.3	-
Immunoglobulin domain	0.2	3.1
Methyltransferase	-	3.2
Methanogenesis	-	3.2
DNA repair	-	3.1
DNA damage	-	3
Necrosis	3	-
3Fe-4S	3	-
S-adenosyl-L-methionine	-	2.9
Anion exchange	2.9	-
Allosteric enzyme	-	2.8
ANK repeat	2.8	-
Archaeal flagellum	2.8	-
Glutathionylation	2.8	-
Arsenical resistance	2.8	-
Motor protein	-	2.7
Quorum sensing	-	2.7
Phosphate transport	2.7	-
Topoisomerase	2.7	-
Periplasm	2.7	-
Queuosine biosynthesis	0.5	2.2
Host-virus interaction	-	2.6
Glutathione biosynthesis	2.6	-
Cobalamin biosynthesis	2.6	-
Chromatin regulator	-	2.5
Viral attachment to host cell	-	2.5
Cytokinin biosynthesis	2.5	-
Cobalt transport	2.5	-
Fibrinolysis	2.5	-
Cilium	-	2.4
Amino-acid transport	-	2.4
Leukotriene biosynthesis	2.4	-
Aromatic hydrocarbons catabolism	2.4	-
Mutator protein	2.4	-
Jasmonic acid signaling pathway	2.4	-
Nucleotide metabolism	-	2.3
Serine protease	2.3	-
Cobalt	2.3	-
Purine metabolism	2.2	-
Nitrogen fixation	2.2	-
Fucose metabolism	2.1	-
Serine/threonine-protein kinase	-	2
Sporulation	2	-
Sugar transport	2	-
Ubl conjugation pathway	0.4	0.5
Immunity	0.3	0.5
Histidine biosynthesis	0.4	0.4
Developmental protein	0.2	0.5
CF(0)	0.2	0.4
Nucleus	0.2	0.4
tRNA processing	0.5	0.1

Stress response	-	0.5
Ligase	-	0.5
Fatty acid metabolism	-	0.5
Chloride channel	0.5	-
Translation regulation	0.5	-
Kinetochore	0.5	-
Glycolysis	0.5	-
Heme biosynthesis	0.5	-
Sterol metabolism	0.5	-
Glycerol metabolism	0.5	-
Wnt signaling pathway	0.5	-
Schiff base	0.5	-
Proteoglycan	0.5	-
Autoinducer synthesis	0.5	-
Myristate	0.5	-
Apoplast	0.5	-
Mitochondrion inner membrane	0.5	-
Glycogen biosynthesis	0.5	-
Tyrosine-protein kinase	0.5	-
Photosystem II	0.1	0.4
Initiation factor	0.2	0.3
Differentiation	0.2	0.3
Secreted	0.3	0.2
Polymorphism	0.3	0.2
Toxin	0.3	0.2
Transport	-	0.4
Heme	-	0.4
Acyltransferase	-	0.4
Iron-sulfur	-	0.4
4Fe-4S	-	0.4
Repressor	-	0.4
Transcription regulation	-	0.4
Cell wall biogenesis/degradation	-	0.4
Plastoquinone	0.4	-
Blood coagulation	0.4	-
Aminoacyl-tRNA synthetase	0.4	-
Hemostasis	0.4	-
Disulfide bond	0.4	-
Tryptophan biosynthesis	0.4	-
Pyrimidine biosynthesis	0.4	-
Innate immunity	0.4	-
Steroid metabolism	0.4	-
GTP-binding	0.4	-
Cytochrome c-type biogenesis	0.4	-
Isopeptide bond	0.4	-
Chloroplast	0.4	-
Monooxygenase	0.4	-
Plastid	0.4	-
Cell projection	0.4	-
Aromatic amino acid biosynthesis	0.4	-
Lipid A biosynthesis	0.4	-
Thiamine biosynthesis	0.4	-
Lipid transport	0.4	-
Pyridoxine biosynthesis	0.4	-
Lipid-binding	0.4	-
Ion channel	0.4	-
Apoptosis	0	0.4
Photosynthesis	0.2	0.2
RNA-binding	0.2	0.2
Hydrogen ion transport	0.3	0.1
Glycoprotein	0.3	0.1
Protein biosynthesis	0.3	0.1
Protein transport	-	0.3
Branched-chain amino acid biosynthesis	-	0.3
Cytoskeleton	-	0.3
Host nucleus	-	0.3
Host membrane	-	0.3
Cell inner membrane	-	0.3
Activator	-	0.3
Transmembrane helix	-	0.3
Fatty acid biosynthesis	-	0.3

Transmembrane	-	0.3
Membrane	-	0.3
Centromere	0.3	-
Ligand-gated ion channel	0.3	-
rRNA processing	0.3	-
Transducer	0.3	-
Lipid degradation	0.3	-
Arginine biosynthesis	0.3	-
Signal-anchor	0.3	-
Oxidation	0.3	-
Spermatogenesis	0.3	-
Ubl conjugation	0.3	-
Cell adhesion	0.3	-
RNA-mediated gene silencing	0.3	-
Microtubule	0.3	-
Tumor suppressor	0.3	-
Hydroxylation	0.3	-
EGF-like domain	0.3	-
Ribosome biogenesis	0.3	-
Riboflavin biosynthesis	0.3	-
Gluconeogenesis	0.3	-
Carbon dioxide fixation	0.3	-
Chlorophyll	0.3	-
ATP synthesis	0.2	0.1
tRNA-binding	0.2	0.1
Thylakoid	0.2	0.1
Cleavage on pair of basic residues	0	0.3
Cell membrane	-	0.2
Lipid metabolism	-	0.2
Phosphoprotein	-	0.2
Purine biosynthesis	-	0.2
Acetylation	-	0.2
Amino-acid biosynthesis	-	0.2
Peptidoglycan synthesis	-	0.2
Cell shape	-	0.2
Endoplasmic reticulum	0.2	-
CF(1)	0.2	-
Lectin	0.2	-
Chromosome	0.2	-
RNA editing	0.2	-
Cytoplasmic vesicle	0.2	-
Voltage-gated channel	0.2	-
DNA condensation	0.2	-
Methylation	0.2	-
Antiviral defense	0.2	-
Hydrogen peroxide	0.2	-
Allergen	0.2	-
Septation	0.2	-
Photorespiration	0.2	-
Olfaction	0.2	-
Histidine metabolism	0.2	-
Glycogen metabolism	0.2	-
Pantothenate biosynthesis	0.2	-
Plant defense	0.2	-
GTPase activation	0.2	-
Thiol protease	0.2	-
Arginine metabolism	0.2	-
Actin-binding	0.2	-
Nickel insertion	0.2	-
Microsome	0.2	-
SH3 domain	0.2	-
Proteasome	0.2	-
Threonine protease	0.2	-
rRNA-binding	0.1	0.1
Ribonucleoprotein	0.1	0.1
Ion transport	-	0.1
Lipoprotein	-	0.1
Lipid biosynthesis	-	0.1
Mitochondrion outer membrane	0.1	-
Golgi apparatus	0.1	-
Nucleosome core	0.1	-

Spliceosome	0.1	-
Cytokine	0.1	-
GPI-anchor	0.1	-
Prenylation	0.1	-
Neurogenesis	0.1	-
Serine protease inhibitor	0.1	-
G-protein coupled receptor	0.1	-
Vacuole	0.1	-
Synapse	0.1	-
Coenzyme A biosynthesis	0.1	-
Protease inhibitor	0.1	-
mRNA splicing	0.1	-
mRNA processing	0.1	-
Cell junction	0.1	-
Ribosomal protein	0.1	0
Elongation factor	0	-

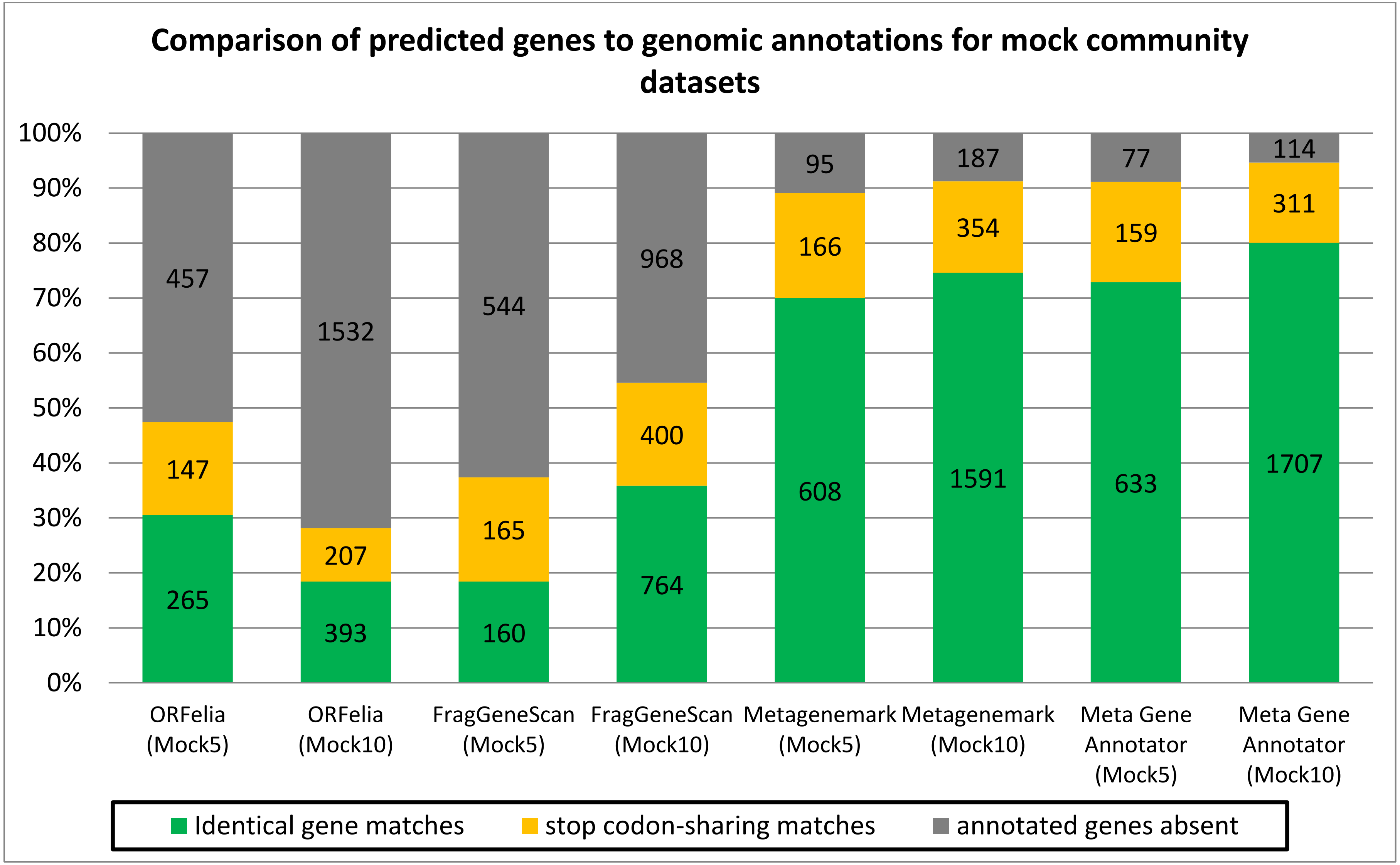

Figure S1. Analysis of gene prediction tools for viral metagenomes. Gene predictions were directly compared to the genomic annotations of source genomes which were used to generate mock community datasets. The total number of predicted genes that were identical to annotated genes are displayed in green. The total number of predicted genes which had the same stop codons, but a varied start codon are displayed in yellow. The total number of annotated genomic genes that were missing from gene predictions is displayed in grey.

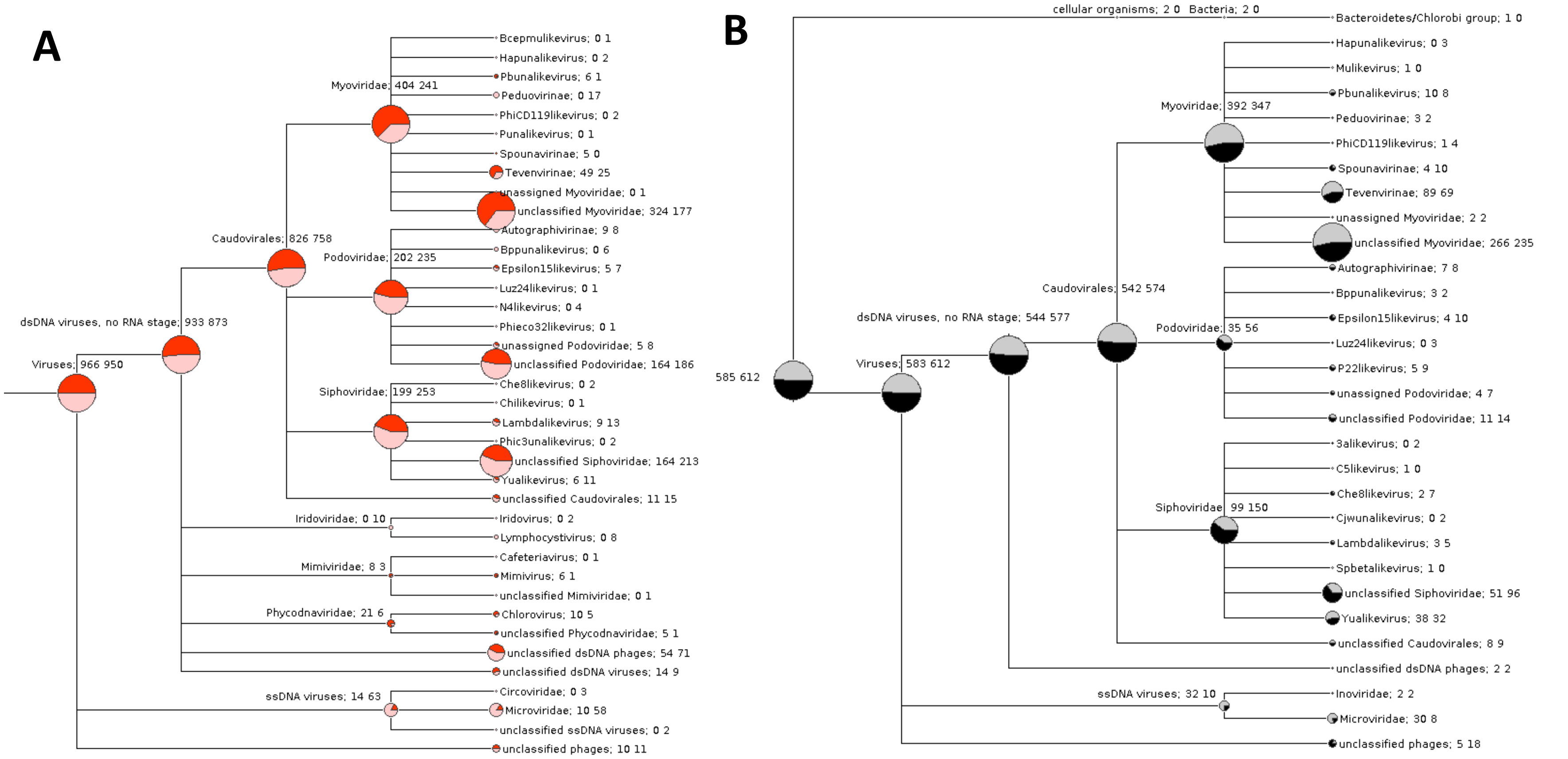

Figure S2 Taxonomic breakdown of *R. odorabile* viral metagenomes. Part A displays normalised taxonomic breakdown of metavirome datasets against viral RefSeq BLAST searches from single read analysis (in pink) and predicted genes from the assembled data (in orange). Part B displays normalised taxonomic breakdown of virome datasets against phage-specific marker and cellular marker BLAST searches from single read analysis (in dark grey) and predicted genes from assembled data (in light grey). MEGAN last common ancestor classification was used to assign taxonomy to reads and genes. Datasets in both parts were normalised against the total number of significant assignments using a minimum bitscore threshold of 80, with taxonomic assignments being made based on 80% consensus of the best BLAST matches. The size of the circles is indicative of the relative composition of the metavirome at each specific taxonomic level (square root scaled). Specific counts of reads that can be assigned to specific taxa are listed to the right of the taxa name (single reads on the left: predicted genes on the right).

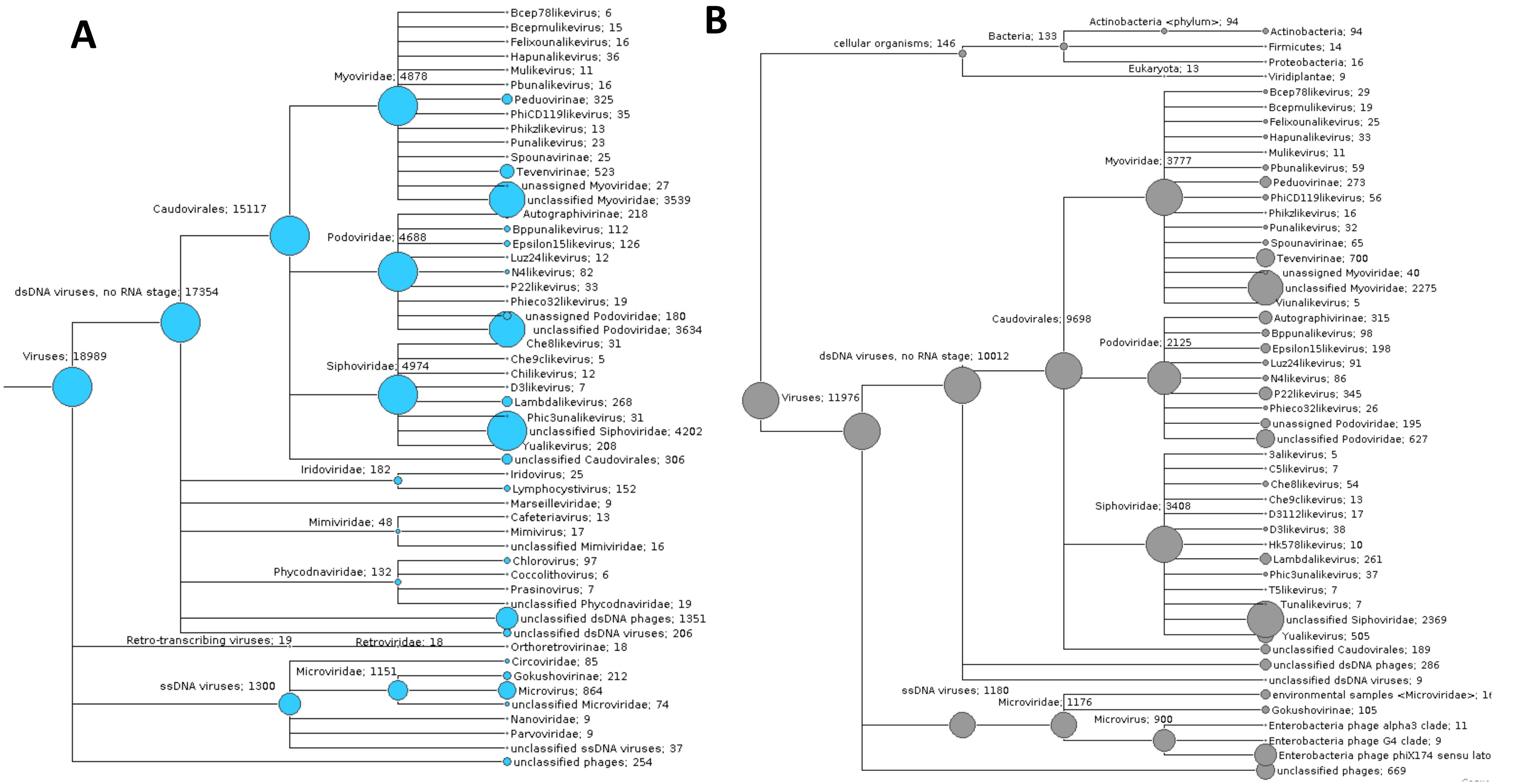

Figure S3. Taxonomic community composition of *P. damicornis* viral metagenomes from single read analysis. Results are based on BLAST analysis of single read data, with taxonomy assigned based on MEGAN last common ancestor classification, using a minimum bitscore threshold of 80, and assignments being made based on a minimum 80% consensus of the best BLAST matches. Viral RefSeq BLAST classifications are observed in part A with composition displayed in blue. Phage-specific and cellular marker BLAST classifications are observed in part B listed in grey. The size of the circles is indicative of the relative composition of the metavirome at each specific taxonomic level (square root scaled). Specific counts of reads that can be assigned to specific taxa are listed to the right of the taxa name.

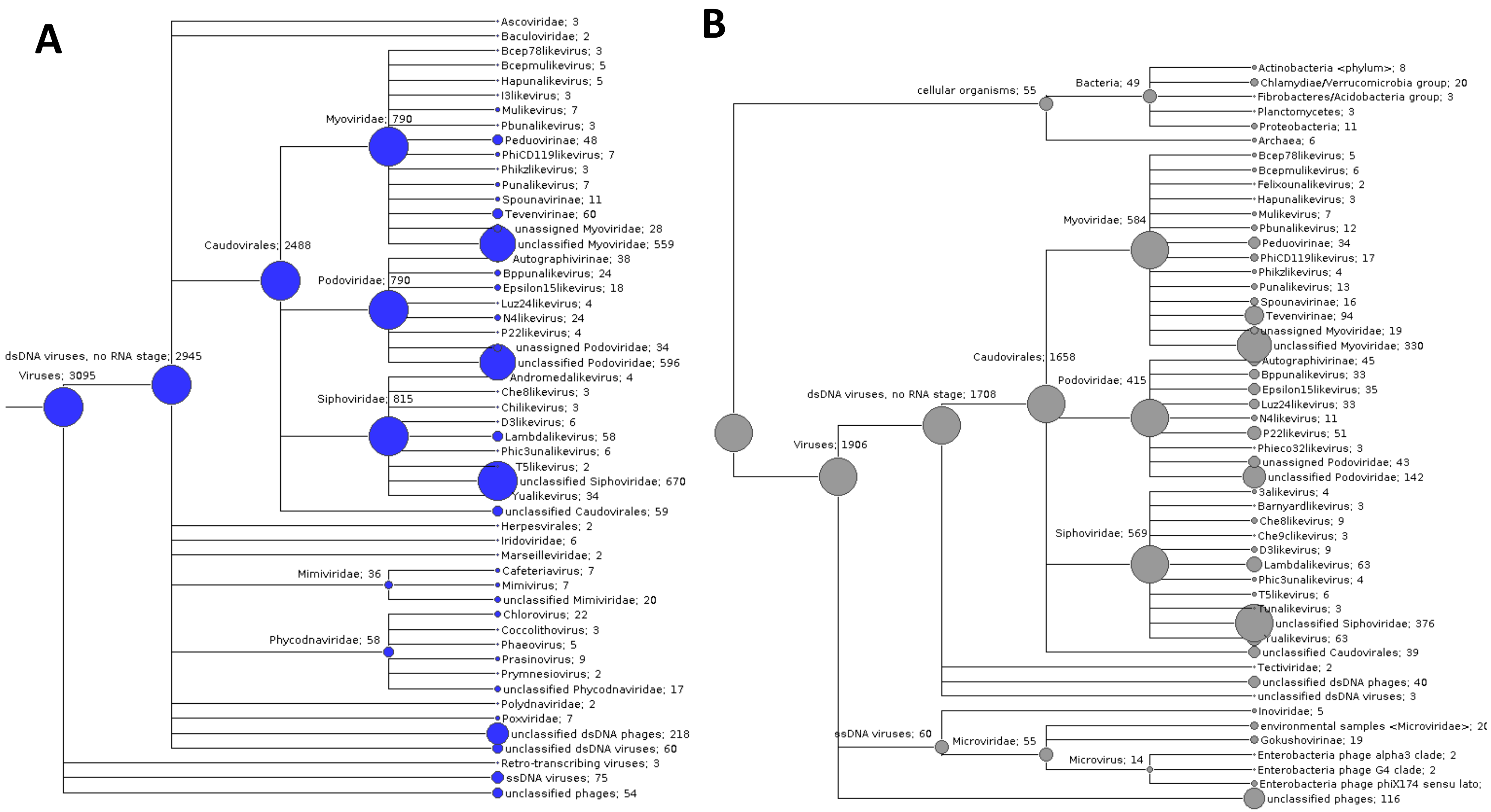

Figure S4. Taxonomic community composition of *P. damicornis* viral metagenomes from MGA predicted gene analysis. Results are based on BLAST analysis of MGA predicted genes from assembled contigs, with taxonomy assigned based on MEGAN last common ancestor classification, using a minimum bitscore threshold of 80, and assignments being made based on a minimum 80% consensus of the best BLAST matches. Viral RefSeq BLAST classifications are observed in blue in part A and viral and cellular marker BLAST classifications are observed in grey in part B. The size of the circles is indicative of the relative composition of the metavirome at each specific taxonomic level (square root scaled). Specific counts of genes that can be assigned to specific taxa are listed to the right of the taxa name.

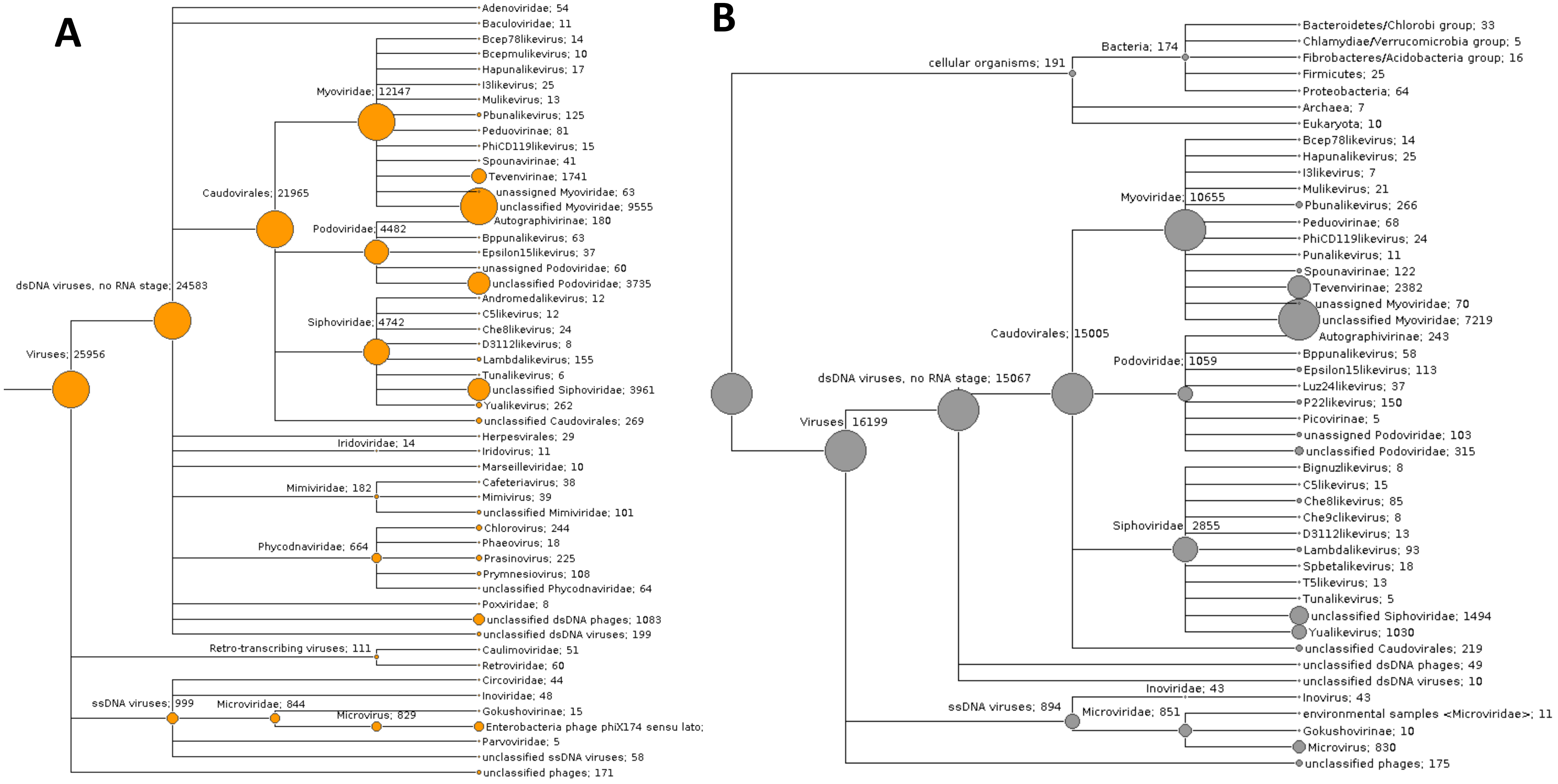

Figure S5. Taxonomic community composition of *R. odorabile* viral metagenomes from single read analysis. Results are based on BLAST analysis of single read data, with taxonomy assigned based on MEGAN last common ancestor classification, using a minimum bitscore threshold of 80, and assignments being made based on a minimum 80% consensus of the best BLAST matches. Viral RefSeq BLAST classifications are observed in part A with composition displayed in orange. Phage-specific and cellular marker BLAST classifications are observed in part B listed in grey. The size of the circles is indicative of the relative composition of the metavirome at each specific taxonomic level (square root scaled). Specific counts of reads that can be assigned to specific taxa are listed to the right of the taxa name.

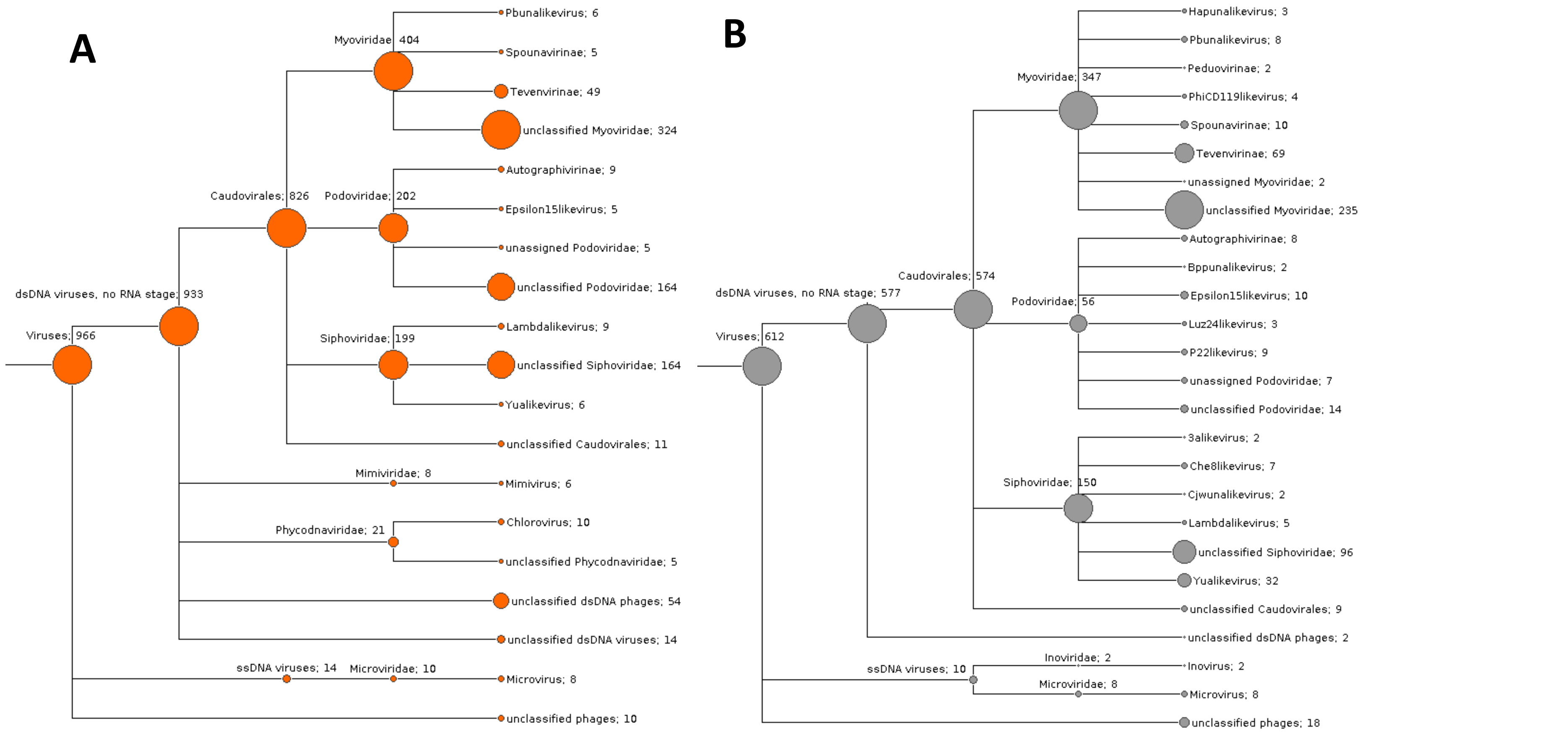

Figure S6. Taxonomic community composition of *R. odorabile* viral metagenomes predicted from MGA. Results are based on BLAST analysis of MGA predicted genes from assembled contigs, with taxonomy assigned based on MEGAN last common ancestor classification, using a minimum bitscore threshold of 80, and assignments being made based on a minimum 80% consensus of the best BLAST matches. Viral RefSeq BLAST classifications are observed in orange in part A and viral and cellular marker BLAST classifications are observed in grey in part B. The size of the circles is indicative of the relative composition of the metavirome at each specific taxonomic level (square root scaled). Specific counts of genes that can be assigned to specific taxa are listed to the right of the taxa name.

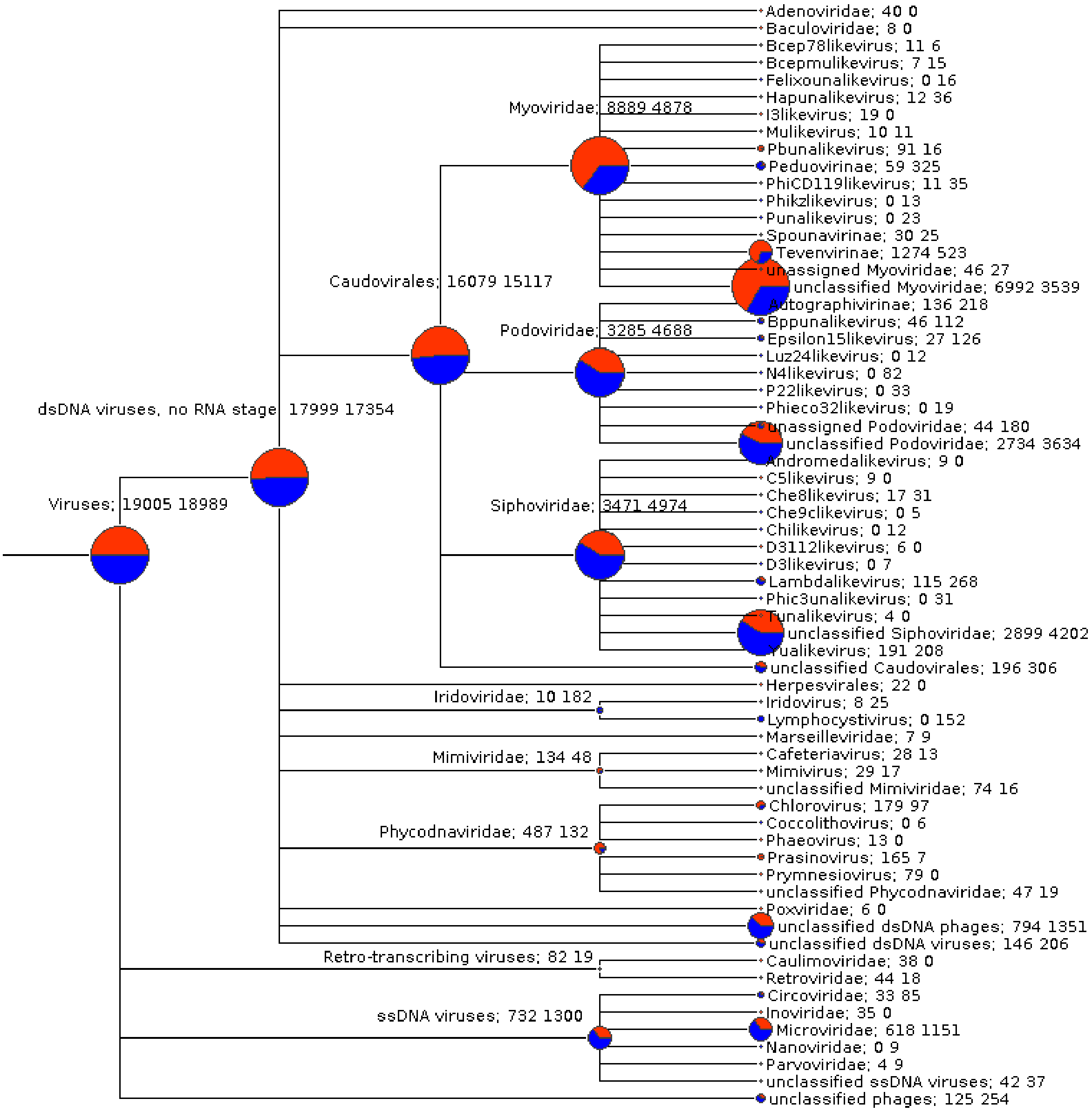

Figure S7.Read-centric comparison of viral metagenomes derived from *P. damicornis* and *R. odorabile*. Results are based on BLAST analysis of single reads from individual metaviromes, with taxonomy of reads assigned based on MEGAN last common ancestor classification, using a minimum bitscore threshold of 80, and assignments being made based on a minimum 80% consensus of the best BLAST matches. Reads from *P. damicornis* are labelled in blue, while genes from *R. odorabile* are labelled in orange. The size of the circles and the proportion of colour is indicative of the relative composition of the metavirome at each specific taxonomic level (square root scaled) for each taxa. Specific counts of reads that can be assigned to specific taxa are listed to the right of the taxa name (*P.damicornis* on the left, *R. odorabile* on the right).
